# Interactions of peptide triazole thiols with Env gp120 induce irreversible breakdown and inactivation of HIV-1 virions

**DOI:** 10.1186/1742-4690-10-153

**Published:** 2013-12-13

**Authors:** Arangassery Rosemary Bastian, Mark Contarino, Lauren D Bailey, Rachna Aneja, Diogo Rodrigo Magalhaes Moreira, Kevin Freedman, Karyn McFadden, Caitlin Duffy, Ali Emileh, George Leslie, Jeffrey M Jacobson, James A Hoxie, Irwin Chaiken

**Affiliations:** 1 Department of Biochemistry and Molecular Biology, Drexel University College of Medicine, 245N 15th Street, New College Building, Room No. 11102, Philadelphia, PA 19102, USA; 2 School of Biomedical Engineering, Science and Health Systems, Drexel University, 3141 Chestnut Street, Philadelphia, PA 19104, USA; 3 Department of Pharmaceutical Sciences, Federal University of Pernambuco, Av. Jorn. Aníbal Fernandes, Cidade Universitária, Recifè-PE, Brazil; 4 Department of Mechanical Engineering and Mechanics, Drexel University, 3141 Chestnut Street, Philadelphia, PA 19104, USA; 5 Department of Molecular Genetics and Microbiology, Duke University, 268 CARL Building, Research Drive, Box 3054 DUMC, Durham, NC 27710, USA; 6 Thayer School of Engineering, Dartmouth College, 14 Engineering Drive, Hanover, NH 03755, USA; 7 Department of Medicine, Perelman School of Medicine, University of Pennsylvania, 295 John Morgan Building, 3620 Hamilton Walk, Philadelphia, PA 19104, USA; 8 Department of Medicine, Drexel University College of Medicine, 1427 Vine Street, 2nd Floor, Philadelphia, PA 19102, USA

## Abstract

**Background:**

We examined the underlying mechanism of action of the peptide triazole thiol, KR13 that has been shown previously to specifically bind gp120, block cell receptor site interactions and potently inhibit HIV-1 infectivity.

**Results:**

KR13, the sulfhydryl blocked KR13b and its parent non-sulfhydryl peptide triazole, HNG156, induced gp120 shedding but only KR13 induced p24 capsid protein release. The resulting virion post virolysis had an altered morphology, contained no gp120, but retained gp41 that bound to neutralizing gp41 antibodies. Remarkably, HIV-1 p24 release by KR13 was inhibited by enfuvirtide, which blocks formation of the gp41 6-helix bundle during membrane fusion, while no inhibition of p24 release occurred for enfuvirtide-resistant virus. KR13 thus appears to induce structural changes in gp41 normally associated with membrane fusion and cell entry. The HIV-1 p24 release induced by KR13 was observed in several clades of HIV-1 as well as in fully infectious HIV-1 virions.

**Conclusions:**

The antiviral activity of KR13 and its ability to inactivate virions prior to target cell engagement suggest that peptide triazole thiols could be highly effective in inhibiting HIV transmission across mucosal barriers and provide a novel probe to understand biochemical signals within envelope that are involved in membrane fusion.

## Background

There is an urgent need for new antiretroviral agents for the prevention and treatment of HIV-1. Most of the currently approved HIV drugs target viral enzymes, in particular reverse transcriptase, protease and integrase [1-4]. In contrast, the number of anti-HIV drugs targeting the entry process is more limited. The proteins involved in HIV-1 entry include gp120 and gp41 organized as a trimer on the viral envelope spike, and both CD4 and a chemokine receptor, either CCR5 or CXCR4, on the cell surface. The fusion inhibitor enfuvirtide (T20) [5] and the CCR5 inhibitor maraviroc [6] are the only currently approved HIV entry drugs for both first-line and salvage therapy [7-9]. T20 targets the N-terminal heptad repeat region of gp41, blocking gp41 conformational changes essential for 6-helix bundle formation and membrane fusion [5]; however, T20 has a relatively short time window to act on the transiently exposed N-helix of gp41 at the cell-virus synapse [10]. In addition, T20 is logistically difficult to administer, as it can only be given parentally, and adverse reactions at sites of injection are common [9,11]. Maraviroc blocks R5-tropic but not X4-tropic HIV-1; thus, clinical use requires co-receptor tropism assays prior to initiating treatment [7-9,12]. Other small molecule entry inhibitors in development include: small molecules against gp120, gp41 and co-receptor [9,13-18]; monoclonal antibodies targeting CD4 [19] and CCR5 [20,21]; and neutralizing antibodies targeting the virion [22-24]. However, none of these latter agents have as yet advanced to first-line clinical use [25-27].

Since gp120 is the first viral protein to interact with the host cell, it is an attractive target for inhibiting infection. We previously identified a peptide triazole class of HIV-1 Env gp120 inhibitors that are highly active on R5-and X4-tropic viruses and exhibit remarkable breadth among different HIV-1 subgroups [28,29]. This inhibition is mediated by binding to a region of gp120 that partially overlaps the CD4 binding site [28-35]. The peptide triazole inhibitors appear to function mechanistically by trapping gp120 in an inactive conformation that is distinct from either the flexible, unliganded conformation or the highly structured, CD4-activated state [28-35]. This conformational entrapment serves to prevent entry prior to virion attachment to CD4 or coreceptor on target cells [30,32]. Recently, a peptide triazole, KR13, was identified that, in contrast to the parental compound HNG156, contained a free sulfhydryl group at the peptide C-terminus [34]. In addition to being a potent inhibitor of R5- and X4-tropic viruses, KR13 induced lysis of pseudotype virions bearing the HIV-1 BaL envelope glycoprotein as determined by release of the p24 capsid protein. This novel effect of KR13 was associated with potent, specific and irreversible inactivation of cell-free HIV-1 virions.

In the study reported here, we sought to characterize more completely the mechanism by which peptide triazoles, and specifically KR13, inactivate and lyse HIV-1 virions. We found that, while all active peptide triazoles tested induced shedding of gp120, only those containing a C-terminal sulfhydryl group induced p24 release. The apparent poration of virions leading to p24 release occurred on replication competent HIV-1 as well as on pseudoviruses bearing HIV-1 Env. Remarkably, lysis was completely inhibited by enfuvirtide, suggesting that the disruption of viral membranes was coupled to physiological activation of gp41 and formation of the 6-helix bundle. We also defined kinetic and biochemical differences between inhibition of viral infectivity and phases of virion disruption. Our findings strongly suggest that the novel virolytic effect induced by KR13 is related to physiological triggering of fusion machinery on the envelope glycoprotein trimer, which in the absence of CD4 or coreceptor engagement leads to disruption of the viral membrane and potent, irreversible viral inhibition.

## Results

### Separation of virus components derived from HIV-1 breakdown induced by peptide triazoles

To probe the effects of peptide triazoles on HIV-1, gradient-purified viral pseudotype particles containing the HIV-1 BaL Env were treated with various peptide triazole variants and then fractionated using iodixanol gradient centrifugation [36]. The density gradient enabled separation of the solubilized proteins from the residual virion fraction. Each gradient fraction was tested for viral infectivity and protein content. Proteins released from virus particles were present in the 6-8% Optiprep fraction (soluble protein fraction), while the intact virions were present in the 18.2-19% Optiprep fraction (residual virion fraction). Solubilized protein and residual virion fractions were evaluated for infectivity and for p24, gp120 and gp41 content (Additional file 1: Figure S1).

### Dose dependence and specificity of KR13-induced virus breakdown

We compared the effects of KR13 and control peptides on HIV-1 infectivity and structure. Peptides included KR13b (KR13 with the thiol blocked with an acetamidomethyl (ACM) group); KR13s, which contains a WX scrambled KR13 amino acid sequence in the gp120-binding IXW pharmacophore [28,29,31]; and the parent peptide triazole HNG156, which contains no free thiol group [31] (Additional file 1: Figure S2). These peptides were chemically synthesized using previously established methods [28,30,31,37]. Peptide binding to gp120 was determined using surface plasmon resonance (SPR) interaction analysis, with K_D_ values for KR13, KR13b, and HNG156 found to be 2.71nM, 6.13 nM, and 13.4 nM, respectively (Additional file 1: Figure S4). Binding analysis of KR13s by SPR direct analysis yielded low and inconsistent dose dependent signals, and no K_D_ was determined in this case. The K_D_ values were determined using BiaEvaluation software using the steady state affinity model. The details of the data fitting used to calculate K_D_ values are explained in the Additional file 2. The activity of the peptides in gp120 binding was further validated by competition ELISA (Additional file 1: Figure S3) with soluble CD4 and monoclonal antibody 17b, the latter of which reacts with a CD4-induced epitope on gp120 that partially overlaps with the coreceptor binding site [38]. These data showed that, with the exception of KR13s, all of the peptide triazole inhibitors used in this study competed with both the CD4 and co-receptor binding sites on gp120. We confirmed that the peptide triazoles in this study did not induce any significant cell toxicity by testing viability of HOS CD4^+ve^ CCR5^+ve^ cells exposed to these inhibitors for 24 hours at 37°C (Additional file 1: Figure S5).

**Figure 1:**
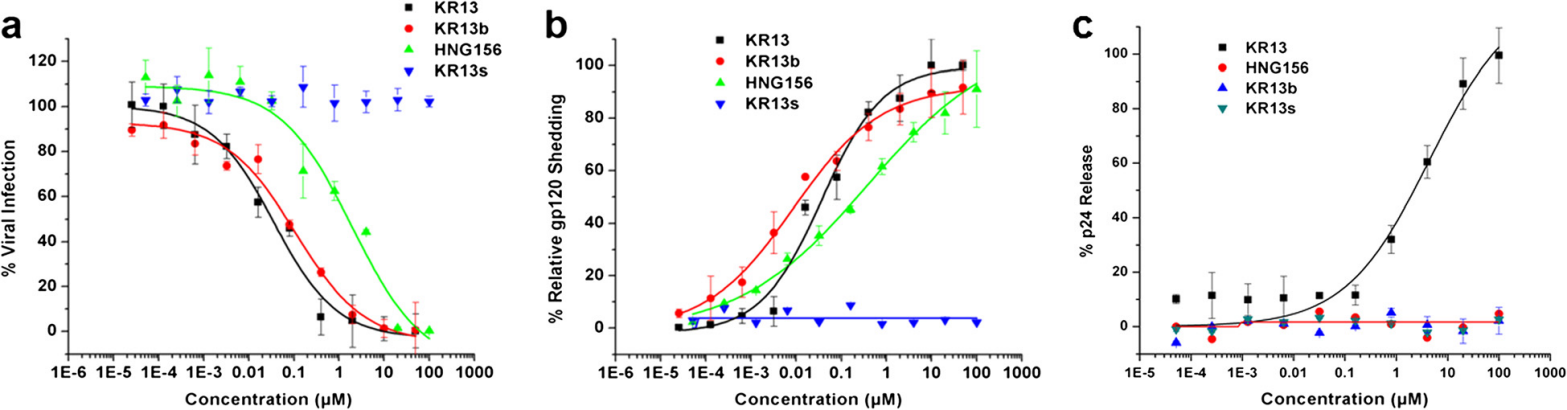
**Dose response of the effects of peptide triazoles on HIV-1 BaL pseudovirus. (a)** Inhibition of cell infection was measured using a single round infection assay. The EC_50_ values are reported in Table 1. **(b)** Relative gp120 shedding was determined for the peptides KR13, KR13b, HNG156 and KR13s using western blot analysis (see Methods section). Electrophoretic bands of gp120 in soluble protein and residual virus fractions were quantified using Image J analysis; IC_50_ values obtained are in Table 1. A low level of gp120 shedding (<5%) was observed with the intact virus used as the negative control. Data were normalized to 100% gp120 shedding observed with 1% triton X treated lysed virus. **(c)** Relative p24 release induced by peptides was measured using ELISA. The data were normalized using untreated virus as negative control (<5% p24 release), and p24 release observed with 1% triton X treated virus was taken as 100% p24 content. The p24 release IC_50_ value for KR13 was 500 ± 80 nM, while the other peptide triazoles exhibited no significant p24 leakage. Sigmoidal curve fits of data were obtained using Origin Pro.8 (Origin Lab). Error bars represent the standard deviation of the mean, n > 3.

Anti-viral effects of the peptide triazoles were initially measured using HIV-1 BaL pseudotyped virions. As shown in Figure 1a, peptides KR13, KR13b, and HNG156 each inhibited infection, with KR13 and KR13b exhibiting the greatest potency, while the sequence-scrambled peptide control KR13s was inactive. Peptide effects on the contents of the virions were determined by treating gradient-purified pseudotyped particles with increasing concentrations of each peptide and determining gp120 shedding and p24 release. The peptides KR13, KR13b and HNG156, but not the KR13s control, caused gp120 shedding (Figure 1b; see Additional file 1: Figure S7 for western blot images). IC_50_ values for gp120 shedding by the active peptides were comparable (Table 1) and similar to IC_50_ values for inhibition of cell infection. In contrast, KR13, but not the other peptide triazoles, induced p24 release (IC_50_ 32 ± 10 nM) (Figure 1c). The samples were normalized to the untreated virus, for which spontaneous p24 release and gp120 shedding were both <5% of the total protein contents as determined by solubilization of the virus particles with 1% triton X treatment (Additional file 1: Figure S7). No inhibition of infection was observed on viruses pseudotyped with either VSV-G or AMLV envelope, indicating that the effects on HIV-1 were highly specific (Additional file 1: Figure S6). All the IC_50_ and EC_50_ values were obtained using sigmoidal plot fit in Origin Pro. 8.

**Table 1:** Potencies of inactivation and breakdown of BaL HIV-1 pseudotyped and replication competent virions by peptide triazoles

**Peptide**	**Viral inactivation**	**p24 release**	**gp120 shedding**
	**EC**_**50 **_**(nM)**	**IC**_**50 **_**(nM)**	**IC**_**50 **_**(nM)**
**HNG156**	803 ± 120	>500,000	500 ± 230
	*5500 ± 1100	* > 200,000	*1800 ± 1000
**KR13**	23 ± 45	500 ± 80	32 ± 20
	*639 ± 71	*4400 ± 930	*1200 ± 930
**KR13s**	>500,000	>500,000	>500,000
**KR13b**	52 ± 35	>100,000	120 ± 43

The extents of loss of cell infection, p24 release and gp120 shedding were measured for KR13, KR13b, KR13s and HNG156 treatment of BaL pseudovirus. * Designates the EC_50_ and IC_50_ values of the viral infection inhibition, gp120 shedding and p24 release, respectively, obtained for fully infectious HIV-1 BaL virions induced by KR13 and HNG156. All of the IC_50_ and EC_50_ values were determined from dose response profiles (Figure 1 and Additional file 1: Figure S12) using Origin Pro.8 (Origin Lab). ± designates the standard deviation of the mean, n > 3.

### Kinetics of HIV-1 breakdown and inactivation

**Figure 2:**
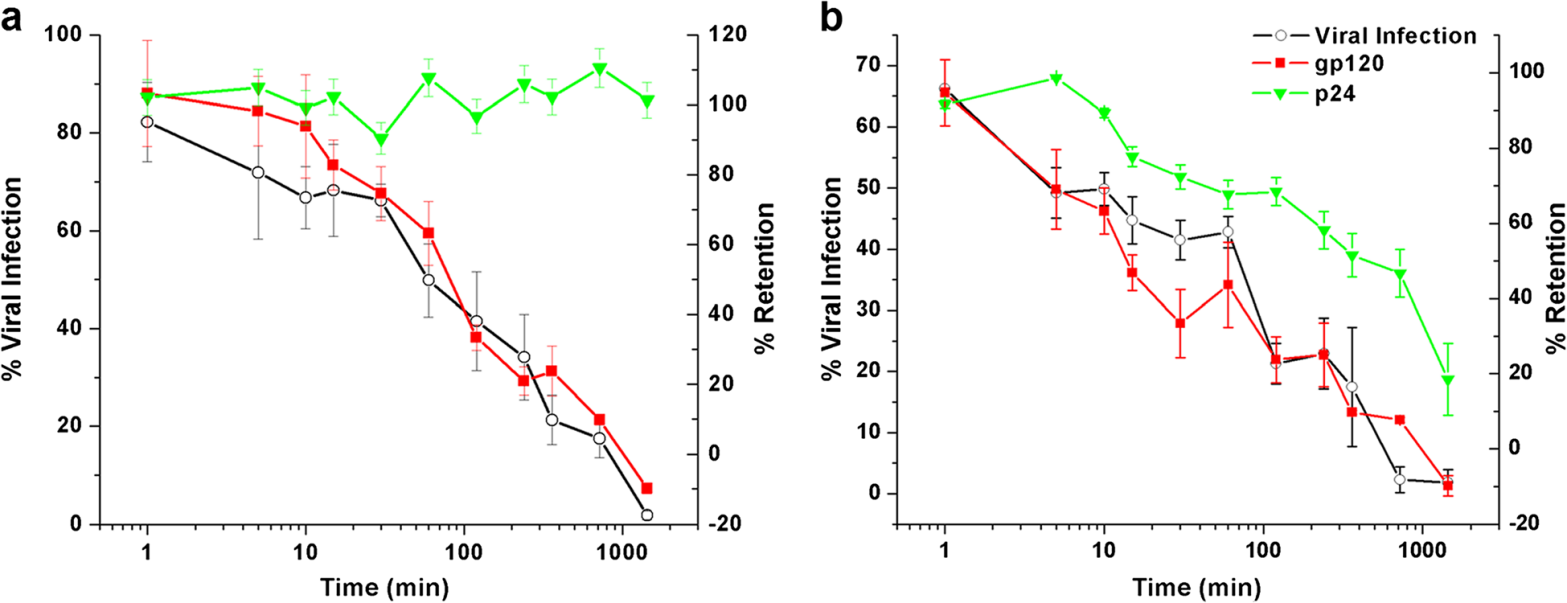
**Time-dependence of viral breakdown by HNG156 (a) and KR13 (b) treatments of HIV-1 BaL pseudovirus.** The % of cell infection retained after peptide treatment is shown on the left y-axes, and the viral protein gp120 and p24 retained in the virus fraction is shown on the right y-axes. All samples were adjusted to the untreated virus as 100% infection and 100% viral protein retention. Each time point had a control of untreated virus, and this was used to normalize each time point of peptide treatment. The concentrations of KR13 and HNG156 were kept constant for each time point at 1 μM and 100 μM, respectively. Untreated controls showed <5% p24 release and gp120 shedding, and <2% loss of cell infection activity. Error bars represent the standard deviation of the mean, n = 3.

We determined the time-dependence of p24 release from HIV-1 BaL pseudoviruses caused by peptide triazoles. Based on IC_50_ values (Table 1), a working dilution of gradient-purified pseudotyped HIV-1 particles was exposed to KR13 (1 μM) or HNG156 (100 μM), with incubation times ranging from 1 min to 24 hours at 37°C. The infectivity of viruses was evaluated, and fractions from post-exposure density gradient fractionation were tested for relative gp120 shedding and p24 release by determining the distribution of protein content between the soluble and virion-containing fractions. As shown in Figure 2, infectivity inhibition and gp120 shedding had similar kinetics for both KR13 and HNG156 peptides. However, p24 release was only induced by the KR13 peptide and was delayed kinetically. Thus, p24 release from viral particles was clearly distinct from effects of peptides on infectivity and gp120 shedding. Each time point was normalized to its own negative control, which was the untreated virus, and to 100% release determined by 1% triton X treated virus. The negative controls showed minimal spontaneous p24 release and gp120 shedding of 5 to 15% from 1 min to 24 hours, respectively. All the IC_50_ and EC_50_ values were obtained using sigmoidal plot fit in Origin Pro. 8, explained in Materials and Methods.

### Retention of gp41 in viral particles treated with peptide triazoles

We characterized the gp41 contents of pseudovirions treated with either HNG156 or KR13. Initial western blot analysis of KR13 and HNG156 treated HIV-1 BaL pseudotype virions were performed using the human anti-gp41 mAb 98–6, which reacts with a linear epitope at residues 644–663 in the gp41 ectodomain. Following either KR13 or HNG156 treatment, gp41 remained associated with virions (Additional file 1: Figure S8), while gp120 was released into the soluble fraction (Figures 1 and 2).

**Figure 3:**
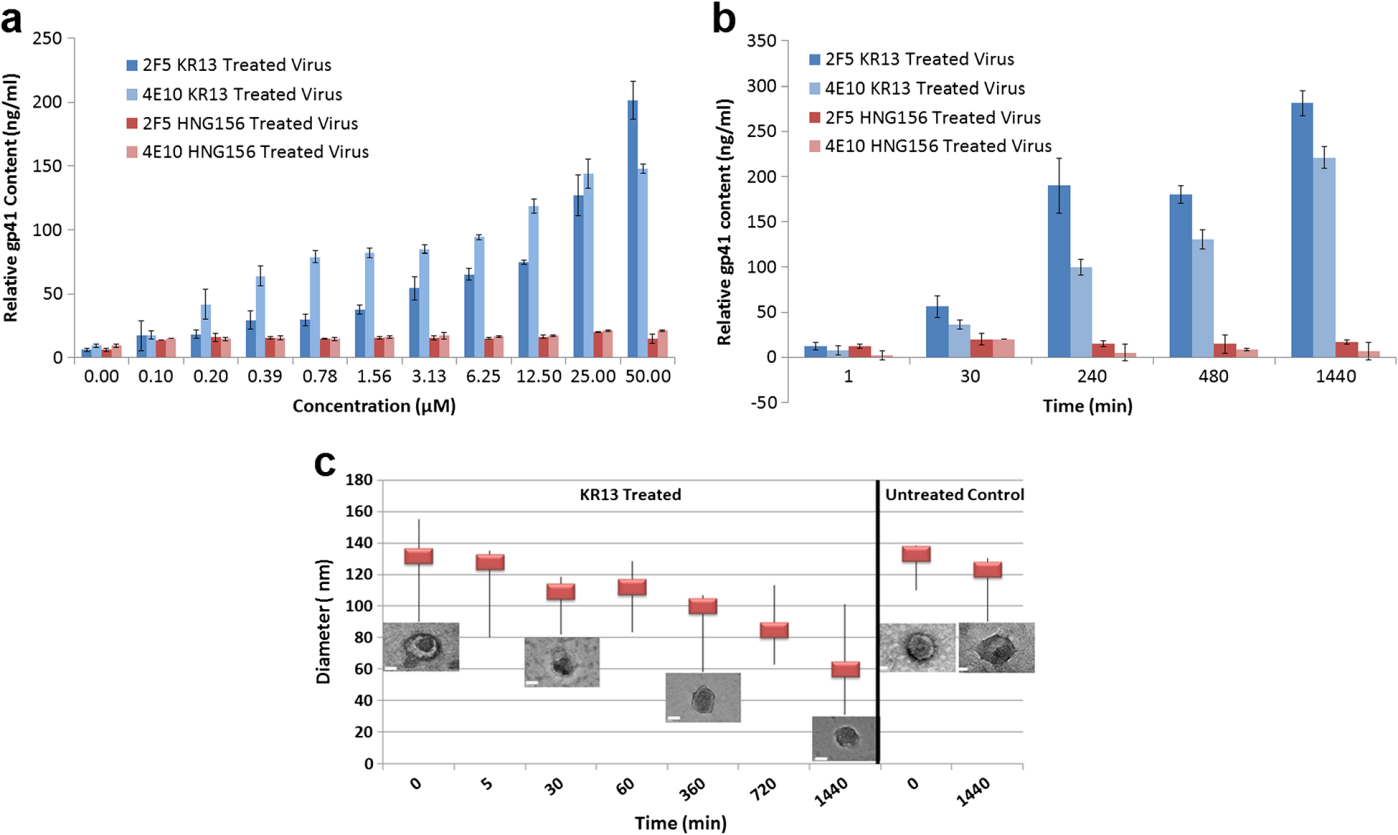
**The gp41 content and morphology of residual virions derived from peptide triazole treatments of HIV-1 BaL pseudovirus. (a)** Reactivity of residual viruses, from 18.2-19% Optiprep fractions, to gp41 antibodies 2 F5 and 4E10 as a function of KR13 and HNG156 concentration. **(b)**. Reactivity of residual viruses to gp41 antibodies as a function of time of treatment with KR13 and HNG156. All samples in **(a)** and **(b)** were normalized to total gp41 content on intact virion. Error bars represent the standard deviation of the mean, n = 3. **(c)** Average diameter of HIV-1 BaL virions untreated and post treatment with KR13 (1 μM) as determined by TEM. The probability distribution box plot shows the average in red and the distribution in bar lines (n = 16). The diameter analysis was conducted using Image J Software. Inset: Representative TEM Images obtained at designated time points, with the scale bar representing 50 nm diameter.

Remarkably, for KR13-treated particles, gp41 was detected only weakly by 98–6 in either soluble or virion-associated fractions. To determine if this effect resulted from a peptide-induced conformational change that blocked exposure of the 98–6 epitope, binding was assessed to conformationally dependent gp41 antibodies 4E10 and 2 F5, which react with membrane-associated MPER epitopes at the base of the gp41 ectodomain. To test the antibody binding, the peptide-treated purified virions were fixed using 0.1% paraformaldehyde in order to maintain gp41 conformation. The fixed virions were evaluated by ELISA as described in the Methods section. On untreated or HNG156-treated virions, MPER epitopes were poorly exposed (Figure 3a and 3b). However, on KR13-treated virions, reactivity to 4E10 and 2 F5 increased in a dose-dependent manner and occurred at concentrations comparable to those required to induce gp120 shedding and p24 release (Figure 3a). This effect was also time-dependent, with kinetics similar to that seen for p24 release (Figure 3b). Thus, while KR13-treatment induced p24 release, gp41 was clearly retained on virions, but in a conformationally altered state associated with loss of 98–6 epitope and exposure of MPER-associated epitopes.

### Morphology of virions treated with peptide triazoles

Transmission electron microscopy (TEM) analysis was conducted to assess the morphology of KR13-treated virions before and after p24 release. Pseudotyped HIV-1 BaL was treated with KR13 (1 μM) from 1 min to 24 hours and samples removed at various time points for TEM imaging. A total of 16 images per sample were taken (Additional file 1: Figure S11), and a probability distribution was plotted to determine the average diameter of the virions pre- and post-KR13 treatment (Figure 3c). Following KR13 treatment, virion diameter was reduced by >50% after 24 hours. In addition and in contrast to the pre-incubation time point when cores were typically condensed and clearly observed, the core morphology of KR13-treated virions at 24 hours was substantially different, with a shriveled and disordered appearance. Similar, although less impressive, morphologic changes were observed at 360 minutes, a time point at which a 50% reduction in virion p24 content was observed (Figure 2). Thus, consistent with biochemical and immunological changes in KR13-treated virions, striking morphologic differences were also evident.

### Inhibition of KR13-induced p24 release by enfuvirtide (T20)

**Figure 4:**
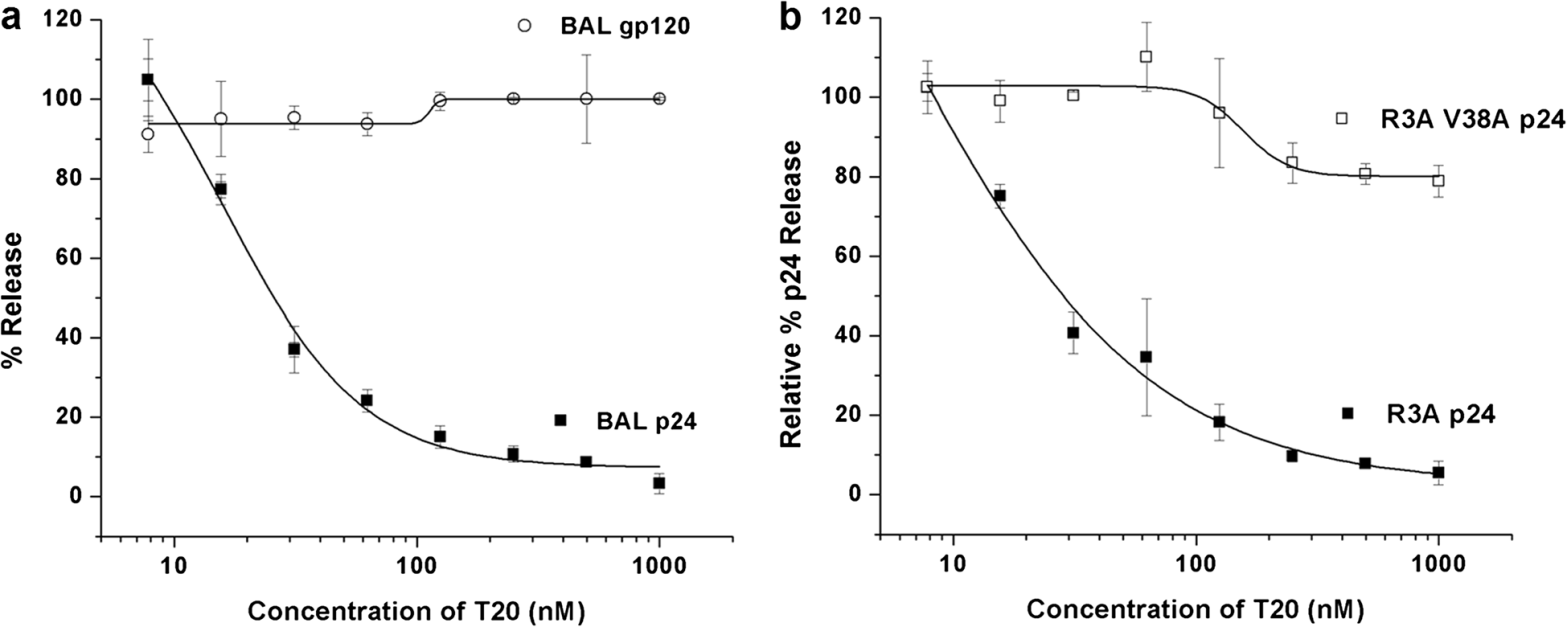
**Inhibition of KR13-induced virus breakdown by the gp41-binding fusion inhibitor T20. (a)** BaL pseudovirus. Virus was treated with 1 μM KR13 in the absence and presence of serial dilutions of T20 starting from 1 μM. Relative p24 release was measured using p24 ELISA by comparing the residual virion (18.2 - 19% Optiprep) and soluble protein (6-8% Optiprep) fractions. Relative gp120 release was measured using western blot analysis by comparing content of gp120 in the analogous residual virion and soluble protein fractions. Western blot values were obtained using Image J analysis of the protein bands. The IC_50_ value T20 inhibition of KR13-induced p24 release was 15.9 ± 4.9 nM using Origin Pro .8 (Origin Lab). No significant effect on the gp120 release in the presence of T20. Error bars represent the standard deviation of the mean, n = 3. **(b)** R3A and R3A V38A pseudoviruses; the latter, mutant virus has been found previously to be resistant to T20 inhibition. Release of p24 was quantified as for BaL pseudovirus in part **(a)**. The IC_50_ of T20 inhibition of peptide induced p24 release from the virus R3A was calculated to be 21.9 ± 5.9 nM. The mutant virus V38A R3A did not exhibit inhibition of p24 release. Sigmoidal fits were obtained using Origin Pro .8 (Origin Lab). Error bars represent the standard deviation of the mean, n = 3.

Given the time- and concentration-dependent release of p24 from pseudotyped HIV-1 BaL virions incubated with KR13, we sought to determine if this effect was associated with well-described physiological changes in Env that occur during fusion. Pseudotyped HIV-1 BaL virions were incubated with KR13 (1 μM) at varying concentrations of T20 for 30 minutes at 37° and p24 release determined. As shown in Figure 4a, T20 produced a striking dose-dependent inhibition in p24 release with an IC_50_ of 15 ± 4.9 nM. T20 did not affect gp120 shedding caused by KR13 (Figure 4a). These data suggest that formation of the gp41 6-helix bundle, which is the target of T20 inhibition [39], is important for KR13-induced disruption in viral particles, as reflected by p24 release. The IC_50_ values were obtained using sigmoidal plot fit in Origin Pro. 8.

To determine specificity of this effect, we evaluated T20 inhibition of KR13-induced p24 release on an HIV-1 that was resistant to T20. A V38A mutation in HR1, previously shown to confer resistance to T20 [40], was introduced into the HIV-1 R3A Env. The wild type R3A and mutant V38A pseudovirions were produced and gradient purified (see Additional file 2). The inhibition of infection by T20 was assessed for HIV-1 R3A and for the V38A T20 resistant mutant of R3A (Additional file 1: Figure S9a). HIV-1 R3A and R3A V38A pseudotype virus particles were incubated with KR13 in the presence or absence of varying concentrations of T20, and p24 release assessed. Both the V38A mutant and the corresponding wild type R3A had infection profiles similar to pseudotyped HIV-1 BaL (Additional file 1: Figure S9b), and each exhibited a similar profile of p24 release (Additional file 1: Figure S9c). However, inhibition of p24 release occurred on parental R3A with an IC_50_ of 5.9 ± 11.9 nM, R3A with the V38A mutation was highly resistant, with an IC_50_ > 1000 nM (Figure 4b).

To confirm that T20 inhibition of p24 release was not caused by artifactual non-specific binding of T20 to KR13, we evaluated interactions of these two peptides by surface plasmon resonance. No significant change in KR13 binding to immobilized gp120 was observed at T20 concentrations up to 10 μM (Additional file 1: Figure S10). Thus, the observed inhibitory effect of T20 on KR13-induced p24 release was specific and likely dependent on the ability of T20 to disrupt formation of the gp41 6-helix bundle. Taken together, these findings strongly suggest that the effects of KR13 on lytic deformation and consequent p24 release are coupled with formation of the 6-helix bundle structure.

### Effects of KR13 on fully-infectious virus

To determine if the effects of peptide triazoles on pseudotyped HIV-1 BaL particles would also occur on infectious viruses, we performed a dose response analysis for viral infection inhibition and p24 release on fully infectious HIV-1 BaL. Virus produced in 293 T cells was incubated with varying concentrations of HNG156 or KR13, and effects on infectivity and p24 release were determined. As shown in Table 1 and Additional file 1: Figure S12, both HNG156 and KR13 inhibited viral infectivity on HOS CD4^+ve^ CCR5^+ve^ cells (IC_50_ values of 0.53 μM and 5.5 μM, respectively), and caused both gp120 shedding (IC_50_ value of 1.2 μM) and p24 release (IC_50_ value of 4.4 μM) (Additional file 1: Figure S12). Thus, the virolytic activity induced by KR13 on pseudotyped HIV-1 virions was also seen with replication competent HIV-1. All the IC_50_ and EC_50_ values were obtained using sigmoidal plot fit in Origin Pro. 8.

### Breadth analysis of peptide triazole thiol induced virolysis

**Figure 5:**
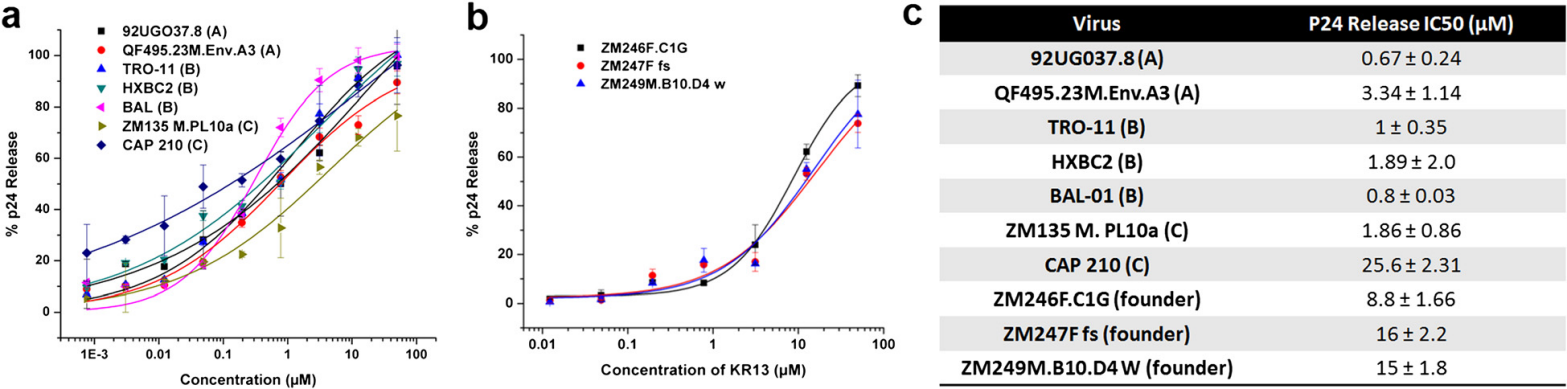
**Antiviral breadth of KR13 induced virolysis.** Dose responses, shown by sigmoidal curve fits, were obtained using Origin Pro. 8 (explained in Methods section) for p24 release induced by KR13 ranging from 1 nM to 50 μM from several clades of HIV-1 pseudoviruses **(a)** and transmitted founder viruses **(b). (c)** Table of the IC_50_ values obtained from **(a)** and **(b)**. Error bars represent the standard deviation of the mean, n = 3.

In order to examine if the virolytic effect induced by KR13 occurs broadly with multiple clades of the HIV-1 virus family, we tested several variants from each of clades A, B and C. We also examined transmitted founder viruses prepared from Env plasmids ZM246F.C1G, ZM247Ffs and ZM249M.B10.D4. From the p24 release effects observed (Figure 5), it is evident that the virolytic effect induced by KR13 indeed occurs broadly, with IC_50_ values ranging from 0.7 μM to 26 μM. This range of action is consistent with the viral infection inhibition breadth analysis conducted by McFadden et al. 2011 for the parent peptide HNG156 [29]. The results obtained show that peptide induced virolysis is conserved and inactivates multiple clades of HIV-1.

## Discussion

Previously, we reported that peptide triazole inhibitors of HIV-1 infectivity target gp120 on the HIV-1 virion and allosterically block CD4 and coreceptor (CCR5/CXCR4) binding sites on gp120 [30,31]. Recently, we discovered that a sub-class of these inhibitors containing a C-terminal sulfhydryl group caused irreversible, cell-independent disruption of viral particles, as shown by release of the capsid protein p24 [41]. In the current study, we sought to more fully characterize the underlying mechanism of these effects. We detected multiple molecular transformations during peptide-induced inactivation and disruption of viral particles. The peptide triazole thiol, KR13, was unique in causing both gp120 shedding and p24 release, and this effect was seen with several clades as well as transmitted founder pseudovirions (Figure 5), showing the breadth of virolytic inactivation. The virolytic effect induced by KR13 was also observed with replication competent, fully infectious HIV-1 BaL virions (Additional file 1: Figure S12). Of note, for the fully infectious virions, the IC_50_ values for peptide induced gp120 shedding as well as virolysis were higher. One possible explanation for this difference is the reduced spike density on infectious virion surfaces compared to the pseudotyped virions. From our data, we observed that the pseudovirions used in this study have spike densities ranging from 60–70 spikes per virion, while the fully infectious BAL-01 had approximately 12–15 spikes per virion. The spike densities were calculated from gp120 content determined by western blot analysis of the virus supernatants as described in Materials and Methods.

**Figure 6:**
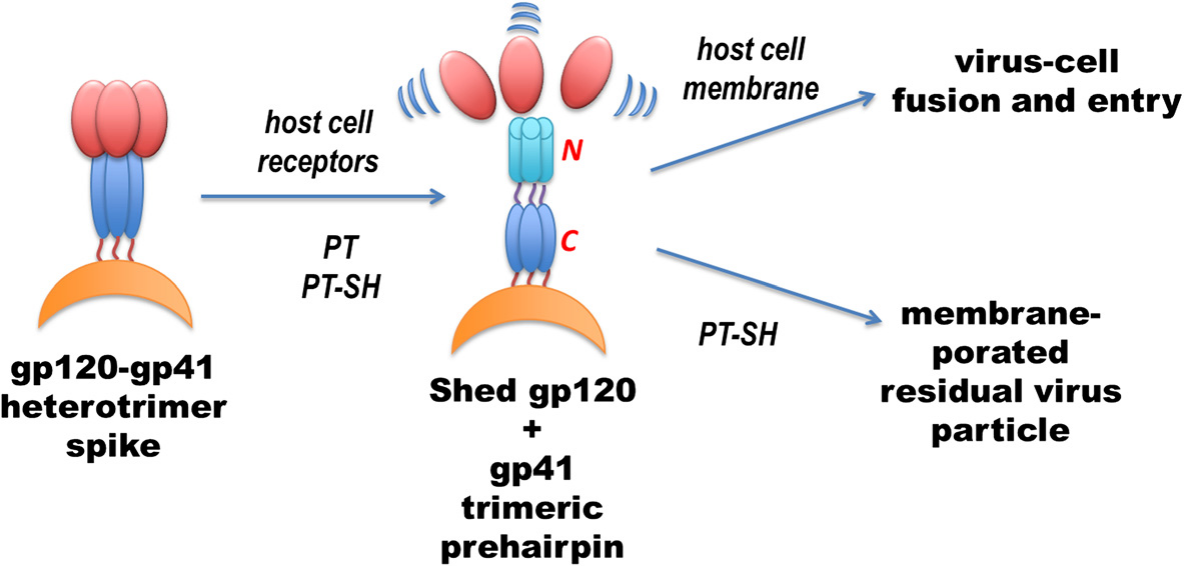
Scheme comparing the HIV-1 deformation steps induced by peptide triazole (PT) and peptide triazole thiol (PT-SH) with gp120 shedding and gp41 trimer prehairpin formation that occurs during cell entry and infection.

Gp120 shedding tracked kinetically with loss of cell infection activity, while p24 release occurred more slowly. The free sulfhydryl group was required for p24 release but not gp120 shedding, as a non-sulfhydryl peptide triazole (HNG156) induced only gp120 shedding as did the KR13b derivative containing a blocked sulfhydryl group. Strikingly, p24 release was potently inhibited by the entry inhibitor, T20, which specifically blocks formation of the gp41 6-helix bundle, on the Env trimer, that is induced following CD4 and coreceptor engagement by gp120 and required for membrane fusion [5]. Moreover, membrane proximal MPER epitopes on gp41, which are characteristically concealed prior to CD4/coreceptor engagement, became highly exposed on viral particles. Overall, gp120 shedding, 6-helix bundle formation, and perturbations in the viral membrane that cause p24 release and gp41 MPER epitope exposure are analogous to physiological events that occur during viral fusion and entry. These findings are consistent with a model in which peptide triazole thiols trigger native structural programs on the HIV Env trimer that are associated with membrane fusion. This view is depicted schematically in Figure 6.

The requirement for the free sulfhydryl group of KR13 in virolysis was demonstrated using synthetic peptide triazole variants. Several peptides in addition to KR13 were examined, including the parent HNG-156 peptide that contained no Cys residue, the KR13b peptide in which the –SH group is blocked and a sequence-scrambled control peptide KR13s (Additional file 1: Figure S2). Both HNG156 and KR13b bound to gp120 and had strong antiviral activities (Figure 1 and Table 1). However, neither of these peptides caused p24 release. Hence, the free Cys-SH group in KR13 is essential for its observed lytic activity. We cannot yet define the mechanistic role of the sulfhydryl group in Env protein disruption. However, Env protein disulfide exchange has been reported to be important in viral entry [42-44]. Hence, it is possible that disulfide exchange may be a component of the KR13-induced gp120 transitions that alter Env spike conformations and, in the absence of a target cell, disrupt the viral membane resulting in breakdown of the virion particle.

We investigated the time dependence of KR13-induced viral disruption by tracking the fate of the viral components gp120, gp41 and capsid protein (p24). We observed (Figure 2) a series of breakdown steps, in which gp120 shedding and loss of infectivity occurred at similar rates, while loss of p24 from the viral capsid occurred more slowly. The difference in rates was evident by a loss of 65-70% of gp120 and infectivity at 30 minutes of KR13 exposure, while only 10% loss of p24 occurred at this time. KR13 treatment also caused a time dependent exposure of the MPER epitope as tracked by the antibodies 2 F5 and 4E10 (Figure 3b). Of note, the membrane-associated MPER epitopes are not well exposed on unliganded viral particles and become transiently exposed during conformational changes associated with entry [45]. Overall, the time dependence of the molecular transitions that occur upon peptide triazole thiol treatment suggests a specific transformation pathway of lytic breakdown that can be related to the organized molecular structure of the virus and changes in that structure that likely occur during the fusion process in virus cell entry.

The presence of a specific time-dependent pathway of virion disruption also fits with observed changes in the physical structure of the virus. The morphology of KR13-disrupted virions was examined by TEM. From the images obtained at different times of KR13 exposure (Figure 3c and Additional file 1: Figure S11), treated virions were smaller, with a >50% reduction in average diameter compared to untreated virions. These results indicate that, rather than resulting in a non-specific and global fragmentation of viral particles, peptide triazole thiol causes a more limited poration, resulting in an intact, although collapsed, particle that releases p24 capsid but retains membrane-associated gp41.

We further evaluated the possibility that this disruption of viral particles and accompanying changes in gp41 might be related to virus-cell fusion by examining the effect of the fusion inhibitor T20. The latter targets the gp41 N-terminal heptad repeat region (N-HR) and blocks 6-helix bundle formation of gp41, an important Env structural transition state in fusion [10]. Indeed, T20 completely inhibited p24 release with an IC_50_ of 15 ± 4.9 nM comparable to its potency in fusion inhibition [46]. No effect was observed of T20 on the KR13-induced gp120 shedding (Figure 4a). Suppression of capsid protein (p24) release by T20 strongly suggests that the formation of the 6-helix bundle prefusion complex may be coupled to peptide-induced poration of the viral membrane and virion inactivation. This possibility was supported by the finding (Figure 4b) that no inhibition of p24 release was seen for an HIV-1 Env bearing a mutation (V38A) that confers resistance to T20 [47,48]. In addition, using surface plasmon resonance analysis, T20 did not interfere with KR13 binding to sensor-immobilized gp120 (Additional file 1: Figure S10), indicating that this effect was not the result of artifactual binding of T20 to the KR13 peptide.

Previous studies have shown that sCD4 [49] and some CD4 mimetic small molecules such as NBD-556 can activate infection in CD4^-ve^ CCR5^+ve^ cells [50]. Since KR13 appears to induce gp41 6-helix bundle formation, and the latter is a required step in cell entry, we evaluated whether KR13 might enhance infection of CD4 negative cells. The same incubation conditions were used as for inhibition of HIV-1 entry into HOS CD4^+ve^ CD4^+ve^ cells. As shown in Additional file 1: Figure S13, no enhancement of infection was observed under these standard conditions. Interestingly, concerning possible effects of peptide triazoles on Env-expressing cells, we have observed using CHO-K1 cells expressing gp160 that KR13 caused gp120 shedding but did not lyse the cells (unpublished results, manuscript in preparation). Hence, the virion structure is important for peptide-induced virolysis.

Because peptide triazoles can inactivate virus by targeting the Env spike leading to irreversible virus breakdown, this class of Env inhibitors may be effective in prevention strategies such as microbicides as novel compounds that specifically inactivate viruses before attachment to host cells [29,51]. In addition, an attractive though at this stage speculative possibility is that the gp41-antigenic properties of virion particles after peptide triazole thiol treatment may make such a process useful for forming attenuated virus products capable of stimulating a neutralizing immune response.

Previous studies have reported several other agents, including peptide NS5A [52], antibodies 2 F5 and 4E10 [53] and sCD4 [54] that lead to HIV-1 breakdown. Peptide triazoles are more HIV-1 specific than NS5A. In addition, viral breakdown triggered by these peptides appears to occur faster than breakdown caused by the antibodies and sCD4 [53,55]. Overall, the current work reinforces the feasibility of novel pharmacologic approaches that can be applied to specifically disrupt viral particles. Furthermore, peptide triazoles can be useful probes to explore poorly understood events, at the viral envelope following CD4 and coreceptor engagement, that lead to alterations of the viral membrane and its fusion to the cell membrane during entry.

## Conclusion

HIV-1 entry, mediated by the viral envelope glycoproteins gp120 and gp41, is an attractive target for preventing infection. Previously, we found that KR13, a sulfhydryl-containing peptide triazole, can bind to gp120, block CD4 and co-receptor binding, inhibit viral infectivity, and physically disrupt viral particles. In this current work, we sought to characterize the mechanism by which these transformations occur. The findings reported here indicate that KR13 peptide triazole initially causes viral inactivation through the release of gp120 followed by subsequent interactions with its free sulfhydryl leading to 6-helix bundle formation, viral membrane disruption, and p24 release. Our data are consistent with the conclusion that KR13 triggers structural changes in the HIV-1 trimer typically associated with viral entry and membrane fusion and that, in the absence of target cells, these changes result in irreversible viral inactivation and lysis. The potent and specific activity of this novel compound and its ability to inactivate virions prior to target cell engagement suggest that KR13 could be highly effective as a microbicide in inhibiting HIV transmission across mucosal barriers as well as a probe to understand biochemical signals required for membrane fusion.

## Methods

Modified Human Osteosarcoma Cells (HOS CD4^+ve^ CCR5^+ve^) engineered to express CD4 and CCR5, receptor and co-receptor respectively, as well as pNL4-3.Luc R-E- backbone DNA, were obtained from Dr. Nathaniel Landau. The HOS CD4^+ve^ CCR5^+ve^ cells were grown in DMEM supplemented with 10% FBS, 2.5% HEPES, 1% Penicillin- Streptomycin, 2% L-Glutamine and 1 mg of puromycin. 293 T cells were obtained from American Type Culture Collection and grown in the same culture medium as the HOS CD4^+ve^ CCR5^+ve^ cells except without puromycin. The plasmids for HIV-1 BaL gp160 and VSV-G Env DNA were gifts from Dr. Julio Martin-Garcia. The antibodies mouse anti-p24, rabbit anti-p24, and the protein p24, were from Abcam. Monomeric YU2 gp120 and sCD4 were produced in-house in 293 F cells following a previously established protocol [29,33]. Fully Infectious HIV-1 (BaL) was a gift from Dr. Michele Kutzler and obtained from Penn Center For AIDS Research (CFAR). The protein gp120 monomer was produced using already-established protocols [33], anti-gp120 was from Alto chemicals. Gp41 protein, enfuvirtide (T20) and anti-gp41 antibodies 4E10, 2 F5 and 98–6 were from NIH AIDS Research and Reference Reagent Program (ARRRP). Enhanced chemiluminescence western blot detection system was from Amersham. O-phenylenediamine (OPD) was from Sigma Aldrich. All other materials were from Fisher Scientific.

### Peptide triazole inhibitors

HNG156 (RINNIXWSEAMM-CONH_2_) and KR13 (RINNIXWSEAMMβAQβAC-CONH_2_), where X is ferrocenyltriazole-Pro, were synthesized by manual solid phase synthesis using Fmoc chemistry on a Rink amide resin at a 0.25 mmol scale [31,41] (Additional file 1: Figure S2). Purity of produced peptides was confirmed by RP-HPLC and MALDI-TOF. Direct binding of the peptide triazole to sensor chip immobilized HIV-1 YU2 gp120 was measured as previously described [28,32] using Surface Plasmon Resonance (SPR) with a Biacore 3000 optical biosensor (GE Healthcare). Steady state analysis was conducted using the method of Morton et al. [56]. Competition assays of soluble CD4 and mAb 17b binding to wild type (WT) YU2 gp120 with increasing concentrations of peptides were carried out by ELISA (Additional file 1: Figure S3). Control peptide triazoles KR13s and KR13b (Additional file 1: Figure S2) were prepared and validated similarly (Additional file 1: Figure S3). Prior to cellular assays using peptide triazoles, we tested for their possible effects on cell viability with HOS CD4^+ve^ CCR5^+ve^ cells after 24 hours of inhibitor exposure. The cell viability was measured using the tetrazolium salt premix reagent, WST-1 from Takara Bio Inc. following the manufacturer’s protocol. The formazan product was measured using the microplate reader at absorbance wavelength 460 nm (Molecular Devices).

### Single-round recombinant luciferase-producing HIV-1 virus-like particles (VLPs) and validation

Recombinant pseudoviruses consisted of the pro-viral envelope plasmid sequence of the CCR5 targeting HIV-1 BaL strain and the backbone sequence of an envelope-deficient pNL4-3-Fluc + env– provirus developed by N. Landau [57]. VSV-G was produced as an envelope control. Pseudotype production followed a modified version of a previously described method for pseudovirus production [58] as explained in the Additional file 2. VLP obtained from culture supernatants was cleared by 0.45 μm syringe filtration and purified on a 6-20% iodixanol gradient (Additional file 1: Figure S1a). Collected fractions were validated for p24 content using the capture ELISA and for gp120 content using western blot detection (Additional file 2). The fractions were validated for infectivity of HOS CD4^+ve^ CCR5^+ve^ cells using the luciferase reporter assay [29] (Additional file 2). Purified virus samples were collected from the 18.2-19% iodixanol fractions of the gradient, aliquoted and stored at -80°C (Additional file 1: Figure S1b).

### Peptide triazole effects on pseudotyped HIV-1 BaL virions

#### ***Dose dependence of peptide-induced viral breakdown***

Serial dilutions of peptide triazole starting from 50 μM were incubated for 30 minutes with working dilution of the purified pseudotyped HIV-1 BaL virus. Control samples included (1) PBS with virus and (2) 1% Triton X-100 with virus. Ten 1 ml fractions were collected from the gradient. Each fraction was tested for p24 content using capture ELISA. High binding polystyrene ELISA plates were coated overnight at 4°C with 50 ng of mouse anti-p24 and blocked with 3% BSA. The blocked plate was rinsed 3 times with PBS-T (PBS and 0.05% Tween 20), and 1 ml gradient fractions were loaded onto the plate using a 1:10 dilution factor with 0.5% BSA in triplicate. After two hour incubation, rabbit anti-p24 was added to the plate for 1 hour, following PBST rinse (3 times, 5 minutes each), and then anti-rabbit IgG fused to horseradish peroxidase (HRP) was added to the plate and incubated for another hour. Following further PBST rinse, o-Phenylenediamine (OPD) was added to the plate and incubated in the dark for 30 minutes. The optical density (OD) was measured at 450 nm using a microplate reader (Molecular Devices). Shedding of the viral envelope gp120 by the peptide triazole was detected in the soluble protein gradient fractions using western blot detection (Additional file 1: Figure S7). Fractions were also tested for viral infection (luciferase reporter system). The western blots were quantified using Image J software, and the values were compared to the lysed virus fraction. Quantified values were analyzed by non-linear regression analysis with Origin Pro.8 (Origin Lab) to determine IC_50_ values. Control peptide triazoles KR13s (scrambled sequence), KR13b (blocked thiol sequence) and HNG156 (parent peptide sequence) were tested for loss of cell infection activity, gp120 shedding and p24 release to determine their effects on viral breakdown and infection inhibition.

### Origin Pro. 8 curve fitting of dose dependence data

Data analysis of dose-dependence measurements performed in this study was conducted by sigmoidal curve fitting using the Origin Pro.8 software. The formula used, which enables a sigmoidal logistic fit, was,

$$y=\frac{A_{1}-A_{2}}{1+{\left(x/x_{0}\right)}^{P}}+A_{2}$$

where A_1_ is the initial value (0), A_2_ is the final value (based on the experimental data), p is the Hill coefficient, x is the concentration of the inhibitor used and x_0_ is the IC_50_ value. The logistic nature of the fitting algorithm allows the p value to float freely. The differences in co-operativity we observe in the plots fits likely arise from complexities of the peptide-virion and virion-cell interactions, a situation which is different than simple protein- protein and protein –peptide interactions.

#### ***Kinetic tracking of transitions during peptide-induced viral breakdown***

The time course of peptide triazole dependent HIV-1 breakdown was evaluated. The respective peptide triazole was pre-incubated with purified virus at a working concentration for times ranging from 1 min to 24 hour at 37°C. The treated samples were then purified on a 6–20% iodixanol gradient as explained above. For each time point, purified virions incubated with PBS at 37°C and alternatively with 1% Triton-X 100 was used as negative and positive controls, respectively. Each fraction was collected and quantified for p24 (capsid protein), gp120 and viral infection as explained above.

### Detection of immunoreactive gp41 on virions post peptide-induced virus breakdown

Residual virus and released protein fractions obtained by gradient purification from KR13 and HNG156 treatments were analyzed for gp41 content. Initially, the presence of gp41 in the treated fractions was detected using western blot analysis with human mAb 98–6 followed by anti-human IgG HRP secondary antibody. Blots were analyzed using the Enhanced Chemiluminescence detection system (Amersham).

We also measured the presence of gp41 epitopes for human mAb’s 2 F5 and 4E10 using an altered ELISA detection method to minimize virus particle disruption [59]. Pseudovirions were treated for 30 min at 37°C with increasing concentrations of either KR13 or HNG156, and samples spun on a 6-20% iodixanol gradient. Virus fractions were collected and fixed with equal volume of 0.1% paraformaldehyde for 30 minutes at 4°C. The treated virus fractions were spun at 16,000 X g for 2 hours at 4°C in an Eppendorf table top centrifuge. Following PBS wash, fixed virions were coated on an ELISA plate at 50 μl per well and incubated overnight at 4°C. The plate was blocked with 3% BSA, and ELISA was used to detect gp41 epitopes with human gp41 antibodies 2 F5 and 4E10 followed by addition of anti-human IgG HRP secondary antibody. This method also was used to assess time-dependent exposure of gp41 epitopes on the pseudotyped HIV-1 BaL virions induced by KR13 and HNG156 treatment at 1 μM constant concentration.

### Analysis of the peptide-treated virions by transmission electron microscopy

Transmission Electron Microscopy (TEM) was conducted to visualize the morphology of the virions treated with KR13. Purified pseudotyped HIV-1 BaL virus and KR13 were pre-incubated from 5–1440 min at 37°C. Following incubation, samples were fixed with 2% glutaraldehyde for 30 minutes at room temperature, and then embedded in Spurr’s low viscosity epoxy medium after acetone washes to dry the virions, slices (100 nm thick) were prepared using an ultra-microtome (Leica EM UC6), loaded onto a holey carbon TEM 200 mesh grid (Electron Microscopy Science) and imaged using the JEM 2100 operated at 120 kV (JEOL, Japan). Sixteen images were taken per sample, and the sizes of observed particles were determined, using Image J software to derive average diameters of the virion particles from TEM images measured from 5 angles.

### Fusion inhibitor enfuvirtide (T20) effects on virolysis

The effect of T20 on virus breakdown by KR13 was assessed. KR13 at 1 μM and serial dilutions of T20 starting at 1 μM were co-incubated with pseudotyped HIV-1 BaL for 30 min at 37°C. Treated virion samples were fractionated on a 6-20% iodixanol gradient (above). Gradient fractions were quantified for p24 using ELISA, and relative p24 release was quantified and plotted using the Origin Pro.8 (Origin Lab). Collected gradient fractions also were analyzed for gp120 shedding using western blot analysis, with detection using primary antibody D7324 and secondary anti-sheep HRP. The bands of the blot were analyzed using Image J software. The IC_50_ values were calculated using Origin Pro.8 (Origin Lab). To further determine whether T20 inhibited peptide-induced virolysis through gp41 interaction, a T20-resistant, gp41-mutated HIV-1 virion was validated and tested for T20 inhibition of KR13-induced virus breakdown. To additionally confirm the specificity of the T20 inhibition, a competition surface plasmon resonance experiment was conducted (Additional file 2). Data were analyzed using Biacore 3000 optical biosensor (GE Healthcare) BiaEvaluation software. Steady state analysis was conducted using the method of Morton et al. [56].

### Inhibition of cell infection and breakdown of fully infectious HIV-1 by peptide triazoles

Fully infectious HIV-1 BaL virions were produced in peripheral blood mononuclear cells (PBMCs) and obtained as a gift from Dr. Michele Kutzler, originally from the CFAR Virology Core, University of Pennsylvania. The replication competent virions were assayed for peptide triazole-induced inhibition of infection, gp120 shedding and p24 release in a BSL-2 facility. Gradient purification of the viruses and p24 release assays were performed as with pseudoviruses (described in Materials and Methods above). Cell infection by virus and its inhibition by peptide triazoles were tested using a modified version of a previously reported p24 ELISA assay [60]. The p24 release assays as well as detection of gp120 shedding were conducted as with the BaL pseudovirions (described in Materials and Methods above). Details are in the Additional file 2.

### Antiviral breadth of peptide induced virolysis

For virolytic breadth analysis, virus envelope plasmids (gp160) of different HIV-1 clades were obtained from the NIH AIDS Reagent and Reference Repository. Further, we obtained the envelope plasmids of founder viruses ZM246F.C1G, ZM247Ffs and ZM249M.B10.D4, originally derived by Dr. George Shaw, as a gift from Dr. James Hoxie. The pseudovirions were produced as described above in the Methods section by the modified Montefiore method, using the Env plasmids obtained as explained above and using the same backbone DNA pNL4-3.Luc R-E-. The produced virions were purified as described above using a 6-20% iodixanol gradient, and further tested for p24 content using ELISA analysis, and for viral infection using luciferase reporter assay system using HOS CD4^+ve^ CCR5^+ve^ cells. The virions were then treated with KR13 (virolytic peptide) for 30 minutes at 37°C at concentrations ranging from 50 μM to 1 nM and then ultracentrifuged at 4°C for 2 hours. The supernatants were collected and tested for p24 content by ELISA analysis as explained above. The % p24 release was calculated relative to the virus treated with PBS taken as 0% p24 release and the virus treated with 0.1% Triton X taken as 100% p24 release.

## Competing interests

The authors declare that they have no competing interests.

## Authors’ contributions

ARB designed and performed the primary experimental studies and analyses and prepared the manuscript; MC contributed to the writing of the paper and helped with experimental design; LDB and DRM synthesized and validated the peptides used; RA assisted with Biacore (SPR) experimental design as well as data analysis; KF assisted with the Transmission Electron Microscopy analyses; KM and CD provided assistance with the data generated for the preliminary anti-viral assays; AE provided guidance and assisted with experimental design; GL provided the plasmids for Env R3A and Env V38A R3A mutant; JMJ provided conceptual input and contributed to the detailed editing of the manuscript; JH provided in-depth experimental design guidance, assisted with manuscript preparation, provided the plasmids of T20 mutants and HIV-1 R3A virus and gave access to the BSL2 facility required for experiments with the fully infectious HIV-1 virus; IC initiated the project and provided guidance for experimental design, interpretation of data and preparation of the manuscript. All authors have read and approved the final manuscript.

## Supplementary Material

Additional file 2 Method details of - Production of single-round recombinant luciferase producing HIV-1 Virus Like Particles (VLPs); Luciferase reporter assay; Western blot detection of gp120 shedding from HIV-1 BaL induced by peptide triazoles; Optical biosensor analysis for direct binding of peptides (KR13, KR13b, KR13s and HNG156) to monomeric gp120; Control optical biosensor analysis to rule out non-specific T20-KR13 interaction; Viral infection inhibition and viral breakdown of fully infectious HIV-1 by peptide triazoles. Click here for file

Additional file 1: Figure S1 Schematic diagram showing purification of intact virus and peptide-triazole derived virus breakdown products by gradient centrifugation. **Figure S2:** Chemical structures and sequences of the peptide triazoles HNG156, KR13, KR13s and KR13b. **Figure S3:** ELISA-derived competition of sCD4 and m17b binding to plate-immobilized gp120 by KR13, HNG156, KR13s, and KR13b. **Figure S4:** Direct binding of KR13, HNG156, KR13b and KR13s using SPR analysis **Figure S5:** Cell viability of HOS CD4^+ve^ CCR5^+ve^ cells in the presence of HNG156, KR13, KR13b and KR13s. **Figure S6:** Inhibition of infection of HOS CD4^+ve^ CCR5^+ve^ cells by recombinant viruses pseudotyped with the envelope for VSV-G and AMLV by the peptides HNG156, KR13, KR13b and KR13s. **Figure S7:** Western blot gel images showing gp120 shedding from HIV-1 BaL pseudovirus as a function of dose of KR13, HNG156, KR13b and KR13s. **Figure S8:** gp41 content measured using mAb 98–6 (anti-gp41) in HIV-1 BaL pseudotype virus after treatment with KR13 and HNG156. **Figure S9:** Infectivity profiles of HIV-1 BaL (WT), HIV-1 R3A (WT) and HIV-1 R3A V38A mutant virions, and infection inhibition by T20 and KR13. **Figure S10:** SPR analysis to test for possible artifactual binding of T20 to KR13. **Figure S11:** Raw TEM images of HIV-1 virions treated with KR13 for 30, 720 and 1440 minutes at 37°C. **Figure S12:** Plots of KR13 induced infection inhibition and virus breakdown of HIV-1 BaL fully infectious, replication competent virus. **Figure S13:** Comparing dose response of the effects of peptide triazoles on HIV-1 BaL pseudovirus induced infection inhibition of HOS CD4^+ve^ CCR5^+ve^ cells and HOS CD4^-ve^ CCR5^+ve^. Click here for file
