# Supplementary material for: Interactions of peptide triazole thiols with Env gp120 induce irreversible breakdown and inactivation of HIV-1 virions
Source: Retrovirology. 2013 Dec 13;10:153. doi: 10.1186/1742-4690-10-153 (PMC3878761; doi:10.1186/1742-4690-10-153)
Supplement: Additional file 2 — Method details of - Production of single-round recombinant luciferase producing HIV-1 Virus Like Particles (VLPs); Luciferase reporter assay; Western blot detection of gp120 shedding from HIV-1 BaL induced by peptide triazoles; Optical biosensor analysis for direct binding of peptides (KR13, KR13b, KR13s and HNG156) to monomeric gp120; Control optical biosensor analysis to rule out non-specific T20-KR13 interaction; Viral infection inhibition and viral breakdown of fully infectious HIV-1 by peptide triazoles. [file 1742-4690-10-153-S2.docx]

**Supplementary Text**

***Interactions of peptide triazole thiols with Env gp120 induce irreversible breakdown and inactivation of HIV-1 virions***

**Production of single-round recombinant luciferase producing HIV-1 virus like particles (VLPs):**

The recombinant virus consisted of (1) the pro-viral envelope plasmid sequence corresponding to the CCR5 targeting HIV-1BaL strain or a VSV (Vesicular Stomatitis Virus) pseudotype and (2) the backbone sequence corresponding to an envelope-deficient pNL4-3-Fluc+env– provirus developed by N. Landau[^1^](#_ENREF_1). 4 µg of envelope and 8 µg of backbone DNA were co-transfected into 293T cells using FuGene 6 as the transfection reagent [^2^](#_ENREF_2). Fourteen hours post-transfection, the medium was changed, and subsequently the VLP-containing supernatants were collected at 48 hours post transfection. The VLP-containing supernatants were cleared using a 0.45 µm syringe filter and purified further by spinning the sample on an iodixanol gradient, using a Hoefer continuous gradient maker, ranging from 6 - 20 % iodixanol, at 30,000 rpm for 2 hours at 4 °C (**Supplementary Fig. S1a**). The collected fractions were validated for p24 content using capture ELISA and for gp120 content using western blot detection as described in the main text. Virions purified on the 6-20% iodixanol gradient exhibited a characteristic distribution profile of p24 and gp120 content (**Supplementary Fig. S1b**), enabling viral (18-19.2% iodixanol) and soluble protein (6-8% iodixanol) fractions to be isolated. The purified virus samples were collected between the 18.2%-19% iodixanol fractions on the gradient, aliquoted and stored at -80°C until further use (**Supplementary Fig. S1b**).

**Luciferase reporter assay:**

A luciferase reporter assay was used to monitor cell infection activity of pseudovirion preparations as well as the inhibition of infection. For the infection profiles, 8,000 cells per well in a 96 well plate were incubated for 24 hours followed by addition of serial dilutions of the gradient purified virus fractions. Forty-eight hours post infection, luciferase activity was measured[^3^](#_ENREF_3). Cells were lysed with 50 µl of Passive Lysis Buffer (Promega) per well for 5 minutes followed by quick freeze/thaw cycles. Luciferase assay was performed using 1 mM D-luciferin salt (Anaspec) as substrate and detected on a 1450 Microbeta Liquid Scintillation and Luminescence Counter (Wallac and Jet). For inhibition of infection assays with the peptide triazoles, infectious dilutions (1 million RLU (Relative Luminescence Units)) of the pre-tested purified virions were pre-incubated with serial dilutions of the inhibitor for 30 minutes at 37°C. HOS CD4^+ve^ CCR5^+ve^ cells, seeded at 8,000 cells per well in a 96well plate, were incubated for 24 hours followed by addition of the pre-incubated inhibitor-virus complex. Forty eight hours post infection, luciferase activity was measured as explained above[^3^](#_ENREF_3). The same method was used in order to measure the effect of the peptides, KR13 and HNG156, on the HOS CD4^-ve^ CCR5^+ve^ cells in order to check for possible enhancement of infection (**Supplementary Fig. S13**). Non-linear regression analysis was used with Origin Pro.8 (Origin Lab) to determine IC_50_ values. All experiments were performed at least in triplicate, and results were expressed as relative infection with respect to cells infected with virus in the absence of inhibitor (taken as 100% infection).

**Cell viability analysis in presence of peptide triazoles:**

Viability of cells in the presence of peptides KR13, HNG156, KR13b and KR13s was assessed using the tetrazolium salt premix reagent, WST-1, from Takara Bio Inc. following the manufacturer’s protocol (**Supplementary Fig. S5**). HOS CD4^+ve^ CCR5^+ve^ cells were treated with increasing concentrations of each peptide ranging from 0.1 µM to 250 µM and incubated overnight at 37°C. Following media change, 10 µl of the tetrazolium salt was added to each sample and incubated for 30 minutes at 37°C. The formazan product was measured using the micro-plate reader at absorbance wavelength 460 nm (Molecular Devices).

**Western blot detection of gp120 shedding from HIV-1 BaL induced by peptide triazoles:**

The HIV-1 BaL pseudovirions were treated with the peptide triazole of interest for 30 minutes at 37 °C, and the fractions were collected from centrifugation in a 6-20% iodixanol gradient. Fractions were boiled with SDS loading buffer for 5 minutes and proteins separated on a 12% SDS non-reducing polyacrylamide gel (NuPAGE – Invitrogen). As a control in the gels, YU2 recombinant gp120 monomer produced in 293F cells was used at 500ng/ml. This control usually runs as a lower molecular weight species compared to the BaL virus gp120. We surmise that this is due to an altered glycosylation pattern for the cell type (293F) used to express the recombinant protein versus the cells (293T) used to produce the BaL virus. The separated proteins in the gels were electrophoretically transferred onto (Polyvinylidene Fluoride) PVDF membrane at 100 mV for 1 hour. The blotted membrane was blocked with 5% skimmed milk in PBST for 1 hour at room temperature. After washing the membrane with PBST, sheep gp120 antibody (D3724) diluted in 5% skimmed milk was added and incubated for 1 hour at room temperature. The bound antibodies were detected by horseradish peroxidase-conjugated anti-sheep IgG secondary antibody followed by the enhanced chemiluminescence detection system (Amersham) according to the manufacturer's protocol (**Supplementary Fig. S7**).

**Optical biosensor analysis for direct binding of peptides (KR13, KR13b, KR13s and HNG156) to monomeric gp120:**

Purified monomeric recombinant gp120 (WT), expressed in 293F cells in house, was immobilized on a Biacore CM5 chip using amide coupling following activation with EDC/NHS (Biacore manual, GE). Briefly, carboxydextran surfaces were activated by injection of a 0.2M solution of 1-Ethyl-3-[3-dimethylaminopropyl] carbodiimide hydrochloride (EDC) and 0.05M N-hydroxysuccinimide (NHS) (both reagents from Pierce). After washing the chip surface with buffer (PBS with 0.005% Tween 20) to remove excess reagents, gp120 diluted in acetate buffer pH 5.5 was injected to the desired coupling level using the manual inject command. Unreacted sites were blocked by ethanolamine and nonspecifically bound protein washed off by a pulse of 10 mM HCl. Approximately 2500 RU gp120 was immobilized on the surface. The first flow cell was derivatized with 2500 RU of anti EGFR mAb, cetuximab, to use as a control surface. All experiments were conducted using ﬁltered and degassed PBS with 0.005% Tween 20 (pH 7.2) at 25°C. Increasing concentrations of peptides, namely KR13, KR13b and KR13s, were injected on the surface from 0.3 nM to 156 nM; CD4 (40 nM) was also injected in duplicate as a control to check for the functionality of the surface immobilized gp120. All injections were done with the kinject command at 100 µL/min and a 250 µL injection volume. The bound peptide dissociated after flowing buffer (PBS + 0.005% Tween-20) at 100 µL/min for 400 s. Ten µl of 10 mM HCl was injected at 100 µL/min flow rate after a 400s dissociation to regenerate the surfaces.

Following double reference subtraction with buffer and control surfaces, (R_eq_) values for each concentration of the peptides were calculated using separate fit analysis for association and dissociation phase for each concentration using BiaEvaluation 4.0 software (GE). R_eq_ values were plotted as a function of concentration of the peptide using a steady state affinity model. Steady state binding level is related to concentration (C) in M and the number of binding sites on the ligand (n, which was 1 in all cases) according to the applied Langmuir equation R_eq_ = K_A_ *CR*_max_/(1+ K_A_*Cn*) for peptide binding using BiaEvaluation 4.0 software (GE) (**Supplementary Fig. S4**).

**Control optical biosensor analysis to rule out non-specific T20-KR13 interaction:**

As part of confirmation of the specificity of the T20 inhibition of HIV-1 lytic deformation by KR13, a competition surface plasmon resonance experiment was conducted. Since T20 requires a small amount of DMSO for adequate solubility, all of the SPR experiments with T20 were conducted in PBS + 0.005% Tween-20 containing 2% DMSO as running buffer. Direct binding of KR13 and T20 respectively was also conducted separately by injecting 0.3 nM to 156 nM of the above peptides in 2% DMSO-containing buffer over the gp120 surface. This was done to monitor the binding of the peptides in the presence of 2% DMSO (**Supplementary Fig. S10**). For KR13/T20 competition experiments, serial dilutions of the T20 (starting from 5 µM) with a constant concentration of KR13 (300 nM) were pre-mixed for 30 minutes and injected over the gp120 surface. Controls included buffer alone, KR13s (300 nM) alone and T20 (5 µM) alone. Data were ﬁt to the Langmuir 1:1 binding equation to estimate steady state aﬃnity in BiaEvaluation version 4.0 (GE) (direct binding) as noted above. The sensorgrams obtained (**Supplementary Fig. 10**) demonstrated lack of interference of KR13 binding to gp120 by T20. This is consistent with lack of nonspecific binding of T20 to KR13.

**Viral infection inhibition and viral breakdown of fully infectious HIV-1 by peptide triazoles:**

Virions were purified using 6-20% iodixanol gradient and p24 content measured using ELISA. The TCID_50_ was determined using Origin Pro.8 (Origin Lab). The working dilution of virus was pre-treated with a serial dilution of KR13 or HNG156 starting from 100 µM for 30 min at 37 °C. HOS CD4^+ve^ CCR5^+ve^ cells seeded, at 8,000 cells per well in a 96 well plate, were incubated for 24 hours followed by addition of pre-incubated inhibitor-virus mixture. After 48 hours infection, p24 content of virions in the supernatant was measured in order to determine the IC_50_ value of viral inhibition. Further, the peptide triazole and cell-free virus were pre-incubated for 30 min at 37°C followed by spinning for 2 hours at 13,600 rpm in a table top centrifuge (Eppendorf) at 4°C. Supernatant was separated and p24 content determined using the p24 capture ELISA (explained in the main Materials and Methods section) and gp120 using western blot analysis (explained in the Supplementary Text above). The extents of viral inhibition and p24 release from fully infectious HIV-1 BaL were also evaluated for control peptide HNG156 (**Supplementary Fig. S12**).

1. He, J. & Landau, N.R. Use of a novel human immunodeficiency virus type 1 reporter virus expressing human placental alkaline phosphatase to detect an alternative viral receptor. *J Virol* **69**, 4587-4592 (1995).

2. Montefiori, D.C. Evaluating neutralizing antibodies against HIV, SIV, and SHIV in luciferase reporter gene assays. *Curr Protoc Immunol* **Chapter 12**, Unit 12 11 (2005).

3. Bastian, A.R. *et al.* Cell-free HIV-1 virucidal action by modified peptide triazole inhibitors of Env gp120. *ChemMedChem* **6**, 1335-1339, 1318 (2011).

4. Morton, T.A. *et al.* Analysis of the interaction between human interleukin-5 and the soluble domain of its receptor using a surface plasmon resonance biosensor. *Journal of Molecular Recognition* **7**, 47-55 (1994).
