# Supplementary material for: Interactions of peptide triazole thiols with Env gp120 induce irreversible breakdown and inactivation of HIV-1 virions
Source: Retrovirology. 2013 Dec 13;10:153. doi: 10.1186/1742-4690-10-153 (PMC3878761; doi:10.1186/1742-4690-10-153)
Supplement: Additional file 1: Figure S1 — Schematic diagram showing purification of intact virus and peptide-triazole derived virus breakdown products by gradient centrifugation. Figure S2: Chemical structures and sequences of the peptide triazoles HNG156, KR13, KR13s and KR13b. Figure S3: ELISA-derived competition of sCD4 and m17b binding to plate-immobilized gp120 by KR13, HNG156, KR13s, and KR13b. Figure S4: Direct binding of KR13, HNG156, KR13b and KR13s using SPR analysis Figure S5: Cell viability of HOS CD4+ve CCR5+ve cells in the presence of HNG156, KR13, KR13b and KR13s. Figure S6: Inhibition of infection of HOS CD4+ve CCR5+ve cells by recombinant viruses pseudotyped with the envelope for VSV-G and AMLV by the peptides HNG156, KR13, KR13b and KR13s. Figure S7: Western blot gel images showing gp120 shedding from HIV-1 BaL pseudovirus as a function of dose of KR13, HNG156, KR13b and KR13s. Figure S8: gp41 content measured using mAb 98–6 (anti-gp41) in HIV-1 BaL pseudotype virus after treatment with KR13 and HNG156. Figure S9: Infectivity profiles of HIV-1 BaL (WT), HIV-1 R3A (WT) and HIV-1 R3A V38A mutant virions, and infection inhibition by T20 and KR13. Figure S10: SPR analysis to test for possible artifactual binding of T20 to KR13. Figure S11: Raw TEM images of HIV-1 virions treated with KR13 for 30, 720 and 1440 minutes at 37°C. Figure S12: Plots of KR13 induced infection inhibition and virus breakdown of HIV-1 BaL fully infectious, replication competent virus. Figure S13: Comparing dose response of the effects of peptide triazoles on HIV-1 BaL pseudovirus induced infection inhibition of HOS CD4+ve CCR5+ve cells and HOS CD4-ve CCR5+ve. [file 1742-4690-10-153-S1.docx]

**Supplementary Figures**

***Interactions of peptide triazole thiols with Env gp120 induce irreversible breakdown and inactivation of HIV-1 virions***

**Figure S1:** Scheme depicting method of purification of intact virus and peptide-triazole derived virus breakdown products by gradient centrifugation. (a) Scheme of location of sample loading and the positions of the 20% and the 6% optiprep fractions after centrifugation. (b) Plot of viral infection, gp120 release and p24 release activities for the iodixanol fractions collected from the gradient. The soluble protein fraction corresponded to 6-8% iodixanol, and the treated virus fraction to the 16-18% fraction, based on the presence respectively of freely soluble p24 and gp120 in the former and viral infection activity in the latter. The controls include virus treated with PBS (intact virus) and virus treated with 1% triton X and boiled for 5 min at 95°C before gradient purification (solubilized proteins).


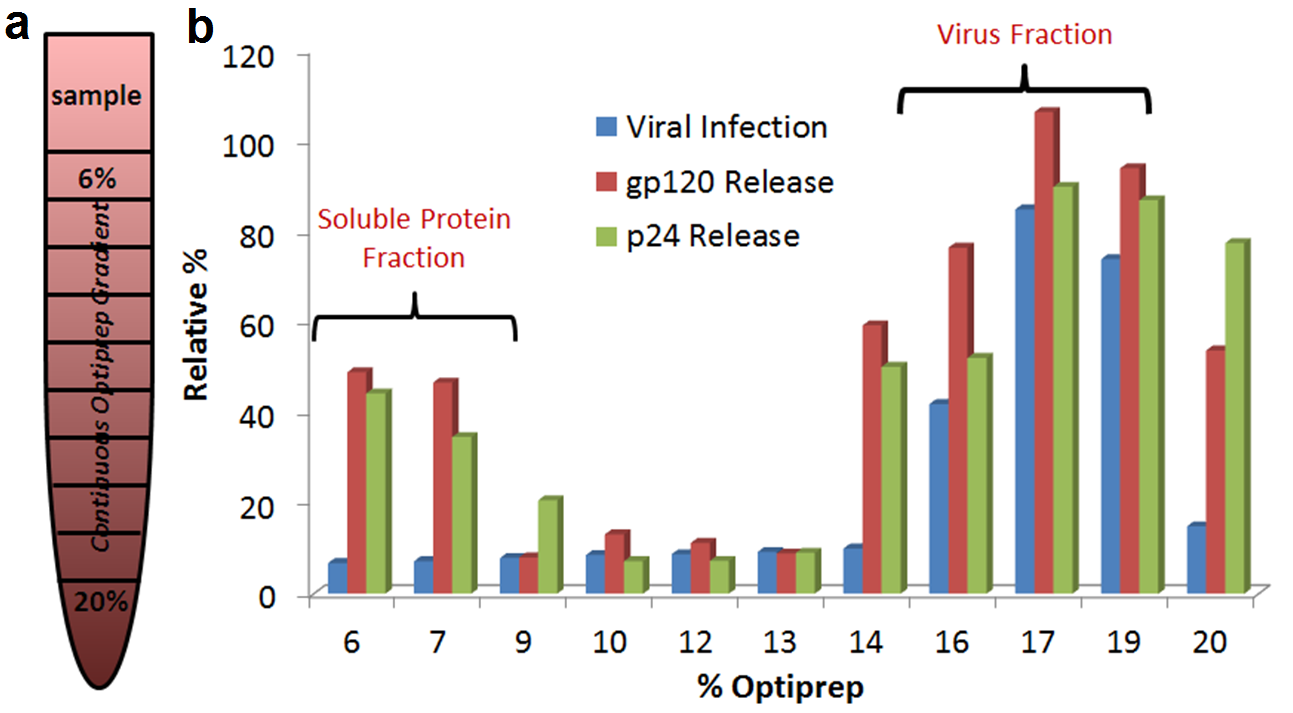


**Figure S2:** Chemical structures (left) and sequences (right) of the peptide triazoles HNG156, KR13**,** KR13s and KR13b. The chemical structures were generated in Chemdraw Pro 13.0. The underlined residues in the one letter coded sequences on the right denote the changes made to the parent peptide triazole (HNG156) to derive the three additional peptide triazoles investigated in this paper. X = ferrocenyl-triazole-Pro and Acm = Acetamidomethyl.


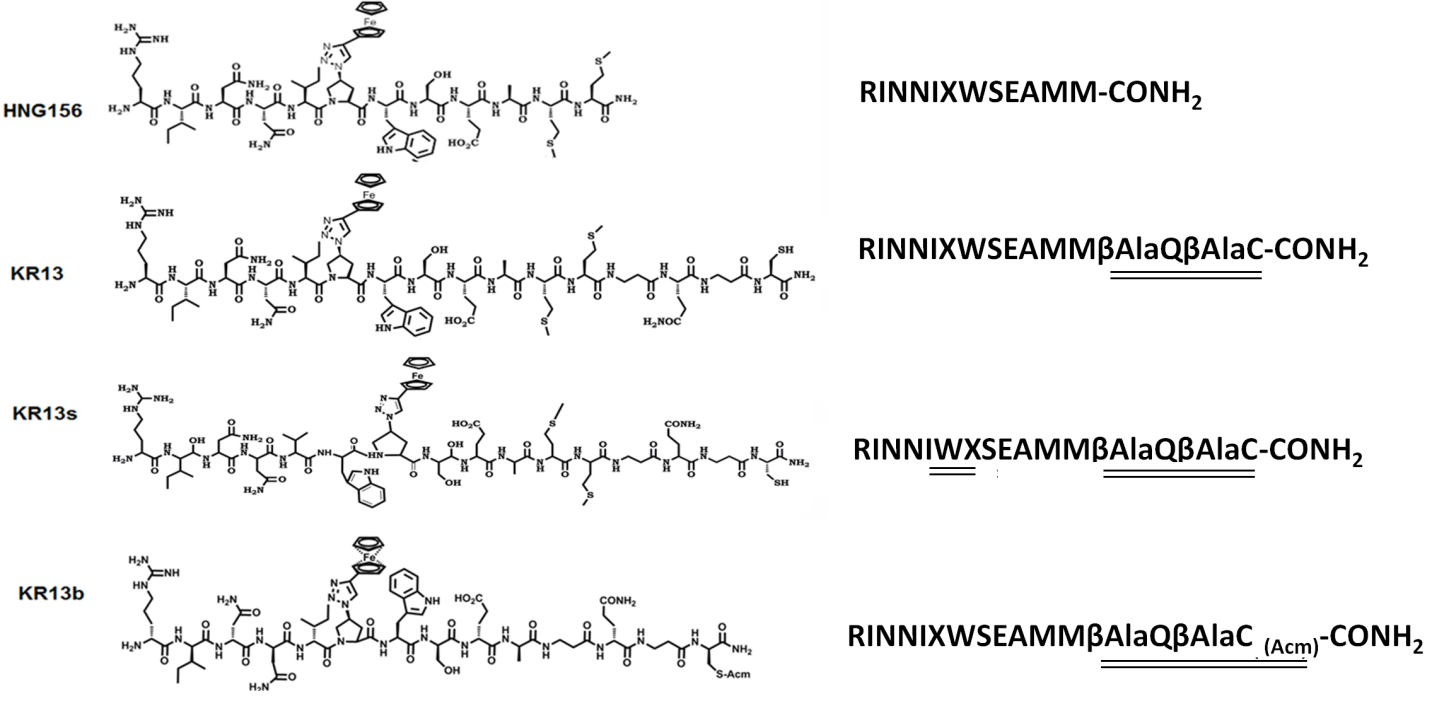


**Figure S3:** ELISA-derived competition of sCD4 (a) and m17b (b) binding to plate-immobilized gp120 by KR13, HNG156, KR13s, and KR13b. The optical density values obtained for sCD4 and 17b binding to the gp120 in the absence of peptide were taken as 100% binding controls. The IC_50_ values calculated for sCD4 competition by KR13, KR13b, HNG156 and KR13s were 22.2 ± 3.2 nM, 15.8 ± 2.9 nM, 12.4 ± 1.2 nM and undetected. The IC_50_ values calculated for 17b competition by KR13, KR13b, HNG156 and KR13s were 20.5 ± 3.5 nM, 415.8 ± 22.9 nM, 165.7 ± 27.6 nM and undetected. Error bars represent the standard deviation of the mean, n > 3.


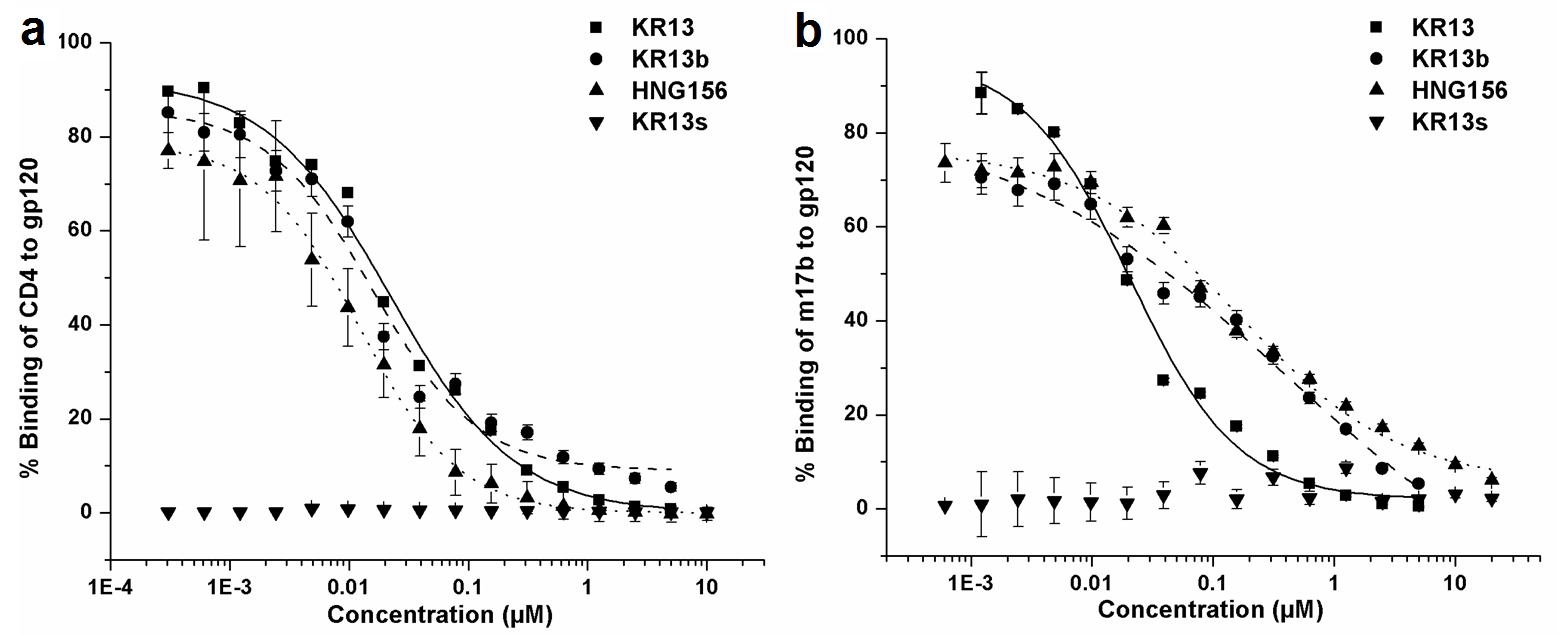


**Figure S4:** Sensorgrams obtained from SPR analysis of direct binding of KR13, HNG156, KR13b and KR13s to gp120. The peptides at concentrations ranging from 0.0003 µM to 0.156 µM were injected at a constant flow rate of 100 µl/min with 250 µl injection volume onto the surface of immobilized YU2 monomeric recombinant gp120. YU2 monomeric recombinant gp120 produced in 293F cells was immobilized on a Biacore CM5 chip at surface density of 2500 RU using EDC/NHS chemistry explained in detail in the Supplementary Text section. The graphs on the left show the sensorgrams obtained post double reference subtraction using the BiaEvaluation software. The graphs on the right show steady state fit analysis of the R_eq_ values for each peptide concentration. The R_eq_ values were calculated using the BiaEvaluation software, with separate k_on_ and k_off_ fit analysis using the Langmuir equation described in Supplementary Text section. Error bars represent standard deviation of the mean, n = 3.

**
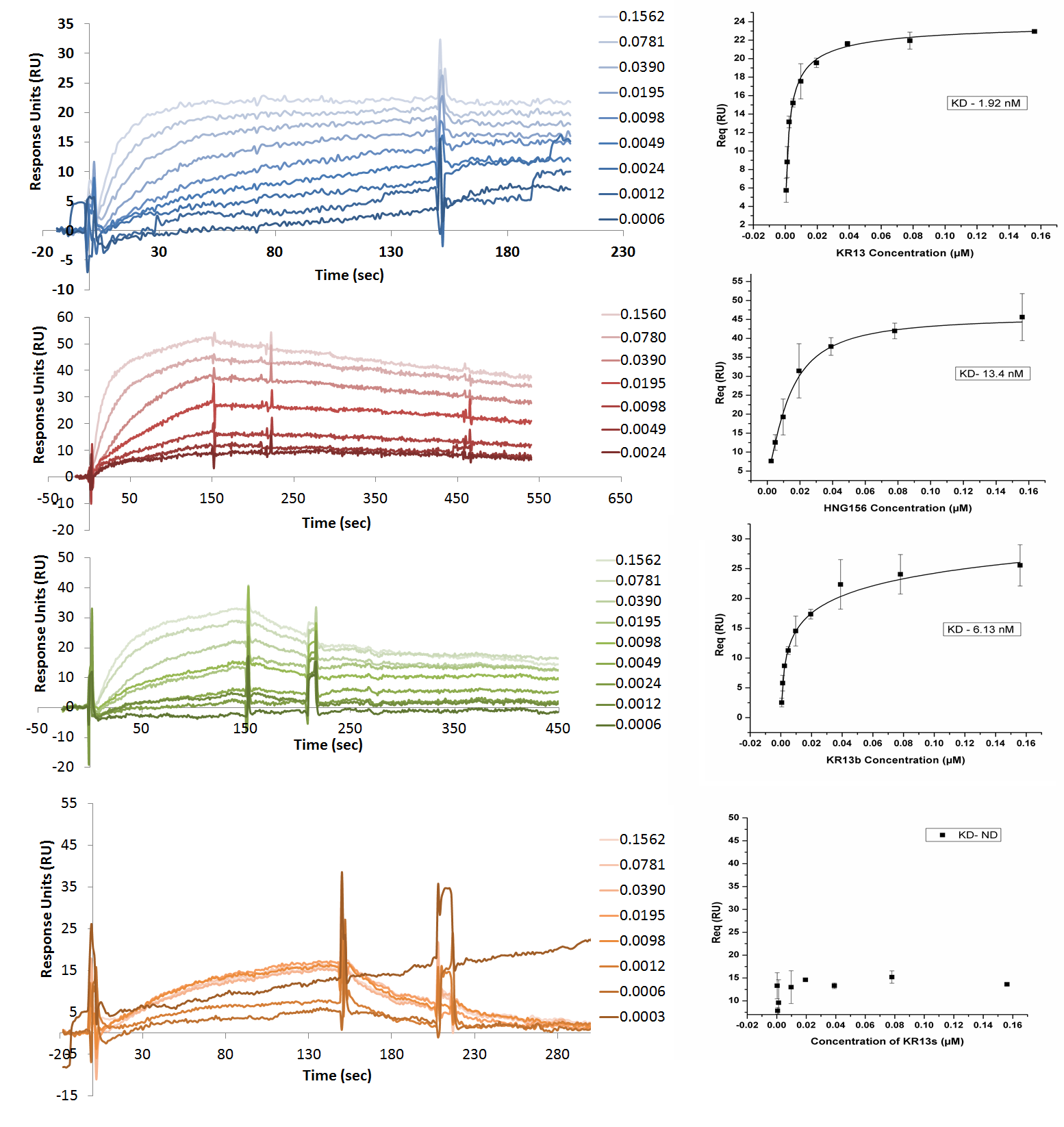
**

**Figure S5:** Cell Viability of HOS CD4^+ve^ CCR5^+ve^ cells in the presence of HNG156, KR13, KR13b and KR13s**.** The samples were tested using the WST-1 assay following manufacturer’s protocol. The viability of HOS CD4^+ve^ CCR5^+ve^ cells in the absence of inhibitor was the 100% control.





**Figure S6:** Inhibition of infection of HOS CD4^+ve^ CCR5^+ve^ cells by recombinant viruses pseudotyped with the envelope for VSV-G (**a**) and AMLV (**b**) by the peptides HNG156, KR13, KR13b and KR13s at concentrations ranging from 250µM to 0.01 nM. The data were normalized to 100% infection activity in the absence of peptide triazole.


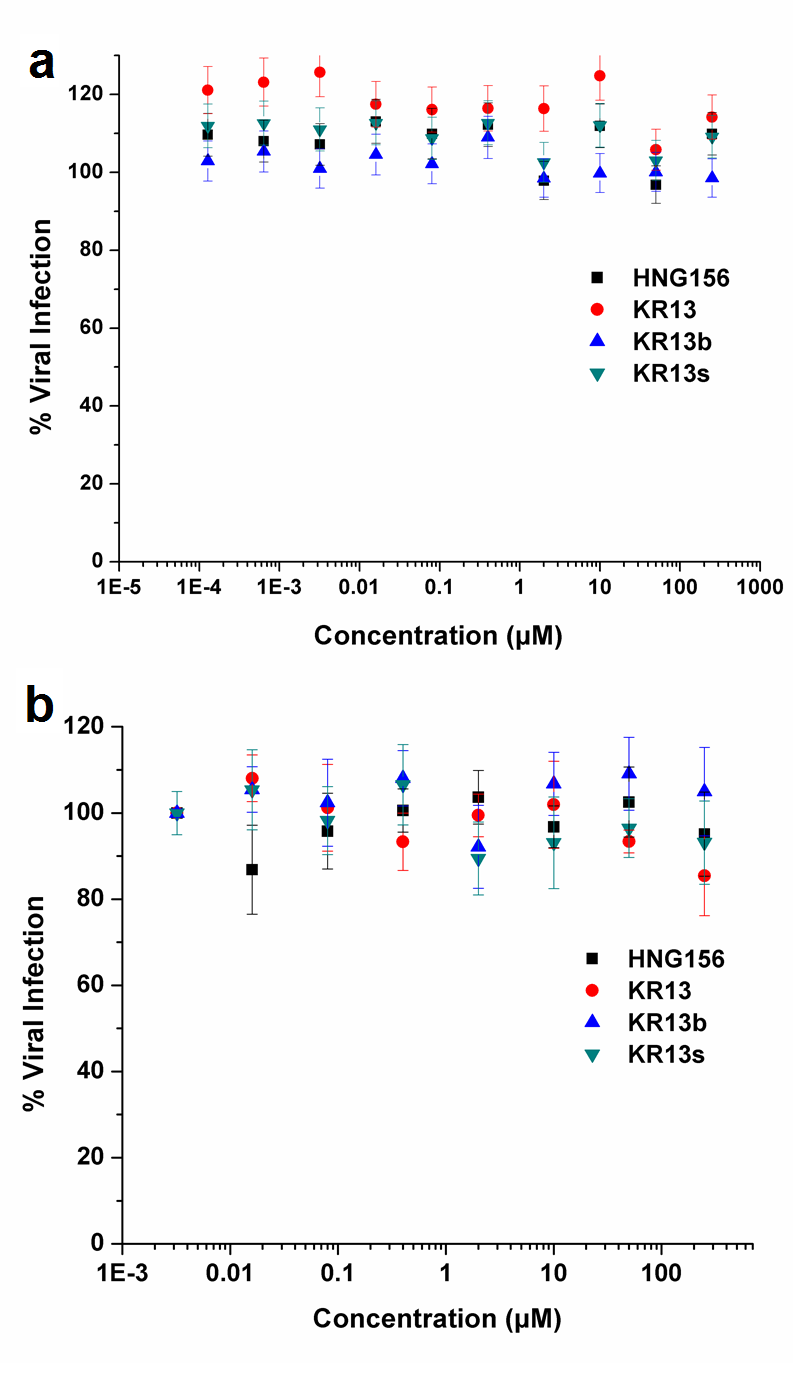


**Figure S7:** Western blot gel images showing gp120 shedding from HIV-1_BaL_ pseudovirus as a function of dose of KR13, HNG156, KR13b and KR13s. The western blots were stained using the primary anti-gp120 antibody D7324 and secondary anti-sheep HRP. Controls shown were intact virus (IV, treated with PBS) and recombinant gp120(200 ng/ml). The gp120 control was the YU2 recombinant gp120 obtained by 293F cell expression and contains monomeric and aggregate bands as observed previously [[1](#_ENREF_1)]. The gp120 from the virions ran at a different size compared to the monomeric gp120 most likely due to a different extent of glycosylation on the surface of these proteins when expressed in 293T cells.


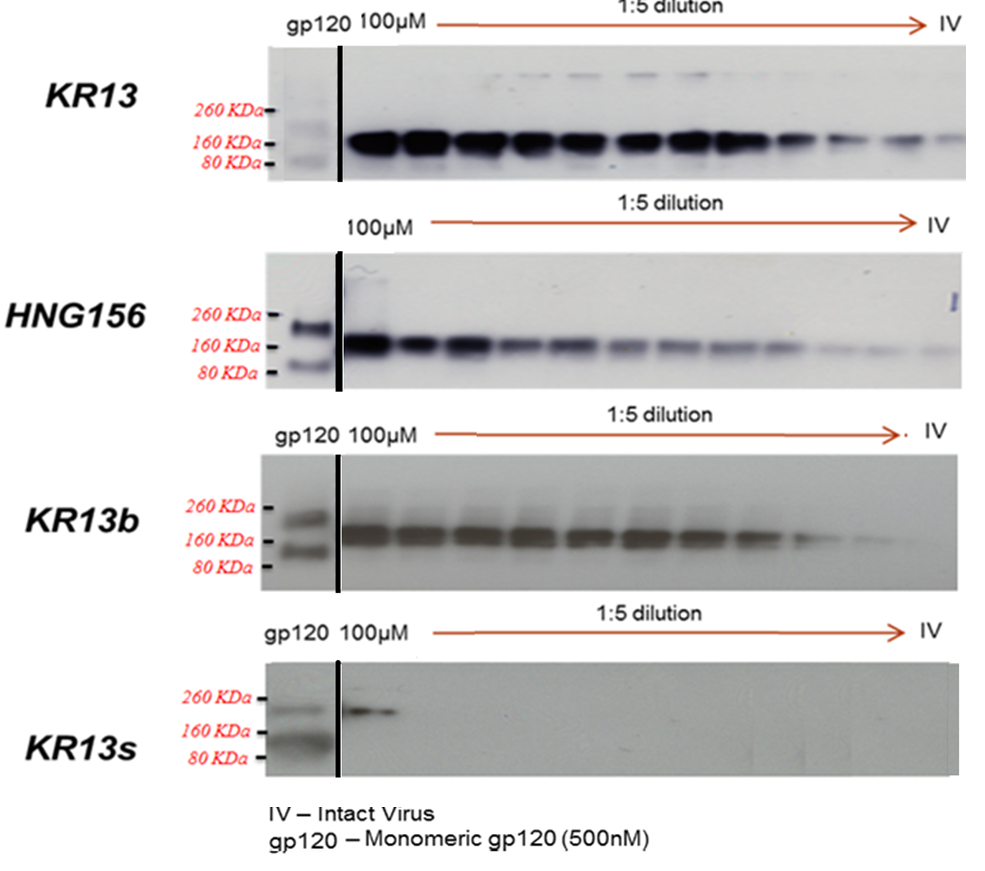


**Figure S8:** Measurement of gp41 content in HIV-1 BaL pseudotype treated with KR13 and HNG156, respectively, as detected by mAb 98-6. Soluble protein (6% Optiprep) and residual virion (18.2 - 19% Optiprep) fractions of the gradient purified, peptide treated virus (HIV-1 BaL pseudotyped) were separated by polyacrylamide gel electrophoresis and transferred to a PVDF membrane. Bands were detected with anti-gp41 mAb 98-6. Band intensities were normalized to total gp41 content present in intact virus control (virus treated with PBS). Error bars represent the standard deviation of the mean, n = 3.


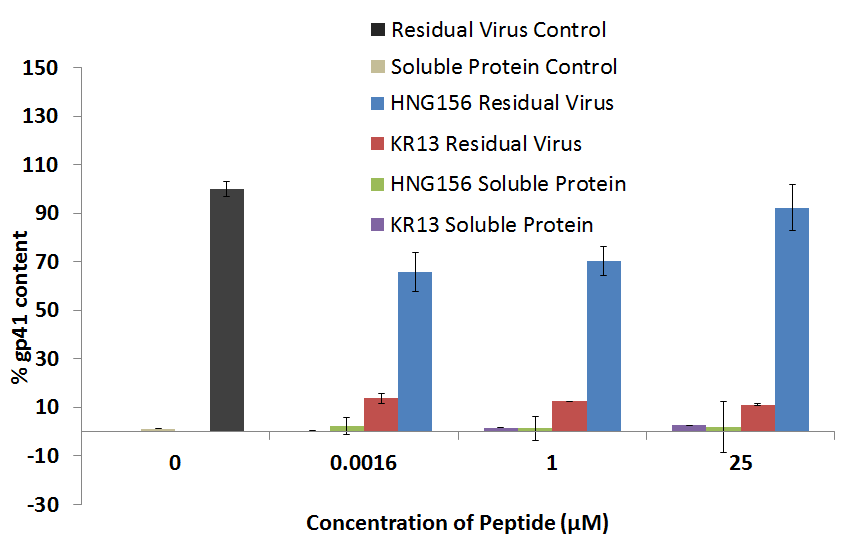


**Figure S9:** Infectivity profiles of HIV-1 BaL (WT), HIV-1 R3A (WT) and HIV-1 R3A V38A mutant virions, and infection inhibition by T20 and KR13. (a) Infection inhibition of BaL, R3A and R3A V38A pseudovirions by T20 had IC_50_ values of 34.5 ± 1.2 nM, 170.8 ± 45.9 nM and 3.3 ± 0.2 µM, respectively. (b) Inhibition of infection of BaL, R3A and R3A V38A pseudovirions by KR13 showed IC_50_ values of 48.7 ± 2.8 nM, 100.5 ± 10.9 nM and 17.8 ± 1.7 µM, respectively. (c) Infectivity profiles of R3A and mutant R3A V38A were determined 24 hours after addition to HOS CD4^+ve^ CCR5^+ve^ cells and expressed in relative luminescence units. Data were obtained using Origin Pro . 8 (Origin Lab). Error bars represent the standard deviation of the mean, n = 3.


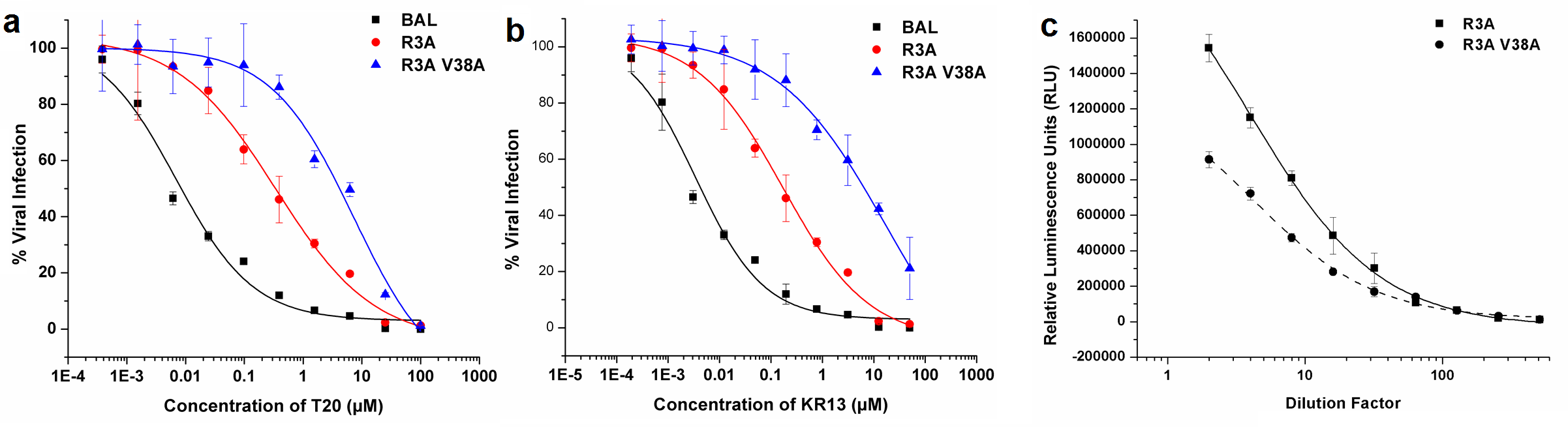


**Figure S10:** SPR analysis to test for possible artifactual binding of T20 to KR13. All the samples were diluted in buffer that contained 2% DMSO and 0.005% tween 20 due to the low solubility of T20 in SPR buffer alone. (a) Sensorgrams for direct binding of KR13 to chip-immobilized soluble WT YU2 gp120. Data show proportionally increasing binding responses to immobilized gp120 at increasing concentrations of KR13. (b) Steady state fit analysis of the R_eq_ values for each concentration of the peptide from (a) calculated using the BiaEvaluation software, with separate k_on_ and k_off_ fit analysis using the Langmuir equation described in Supplementary Text section (c) Sensorgrams for direct KR13 binding to chip-immobilized gp120 in the presence of T20. Samples of T20 (0 µM to 5 µM – shown in the legend) and constant concentration of KR13 (300 nM) were pre-mixed (30 min) and injected into the sensor flow cell. The binding profiles were observed on the same WT monomeric gp120 chip as in (a). As a baseline control, PBS (Buffer) alone was injected, T20 at 5 µM and KR13s at 300 nM were used as a negative controls. Data show lack of significant dose-dependent effect of T20 on KR13 binding to gp120. The negative control surface used was chip immobilized with non-specific EGFR antibody cetuximab. All curves were plotted using Biaevaluation 4.0 software (GE).


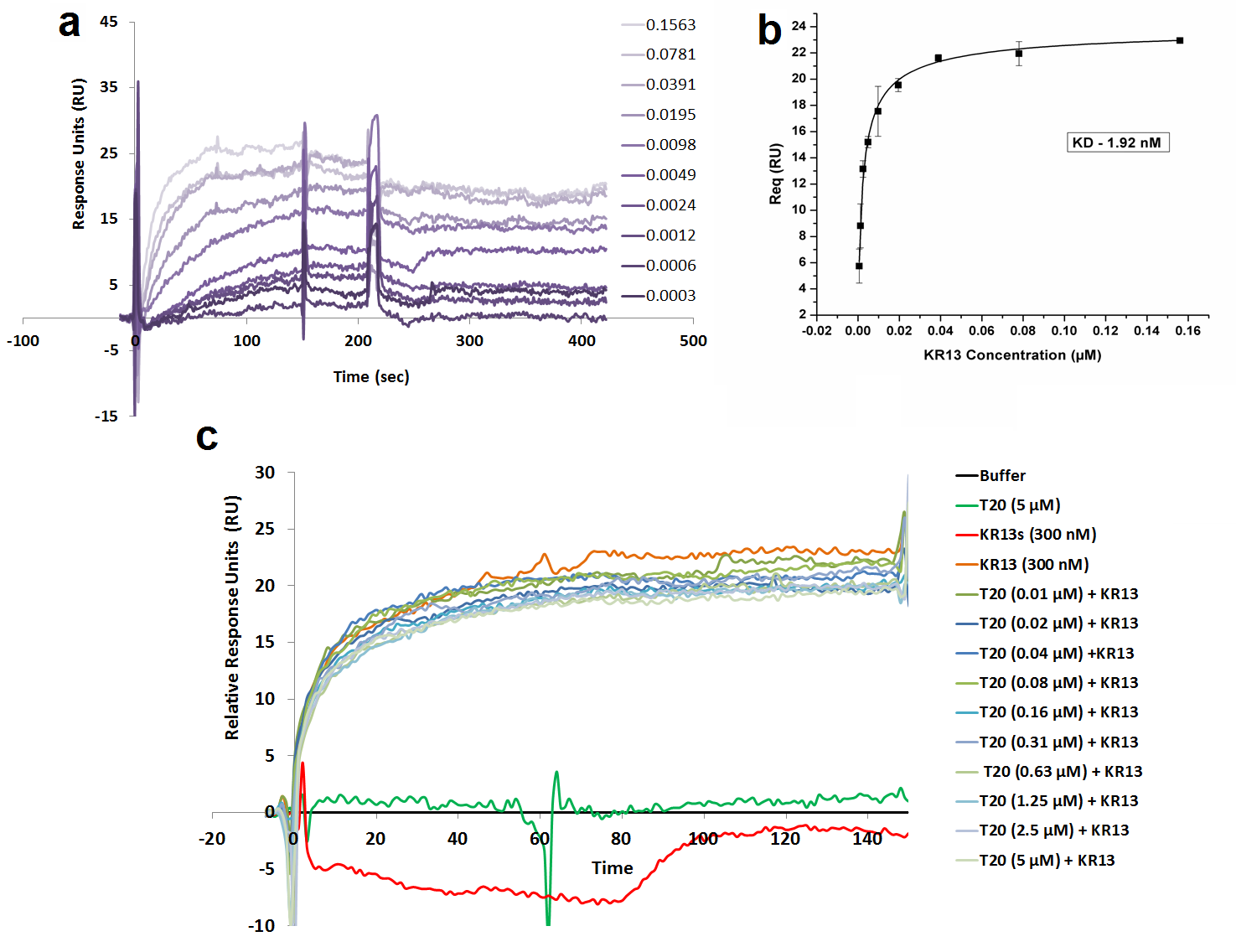


**Figure S11:** Raw TEM images used to determine the diameter distributions in Figure 3. The intact virus field was obtained for viruses treated with PBS for 30 minutes at 37 °C before fixing the virions with 0.1% paraformaldehyde and loading on epoxy for TEM analysis. Separate samples of virions were treated for 30, 720 and 1440 minutes with KR13 at 37 °C before fixing with paraformaldehyde. The epoxy samples were cut into 100 nm thick slices using a Leica EM UC6 Cryo-ultramicrotome and loaded onto a holey carbon TEM 200 mesh grid for TEM analysis. The grids were stained with 1% uranyl acetate and loaded onto the JEM 2100 microscope system (JEOL, Japan) operated at 120 kV.


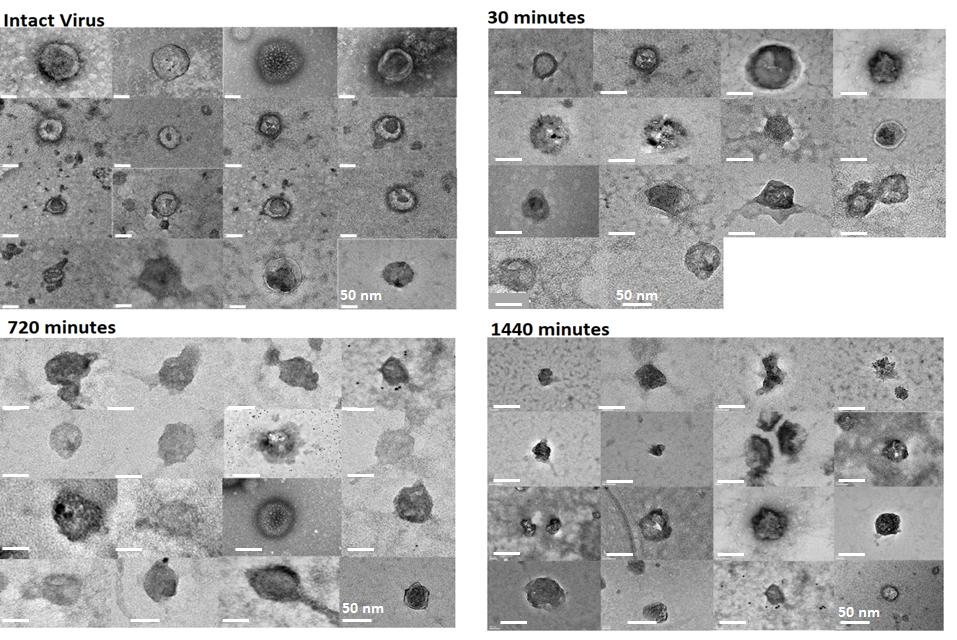


**Figure S12:** Inactivation (a) and virion breakdown (b and c) of HIV-1 BaL fully infectious virus by KR13 and HNG156. The EC_50_ values for inhibition of infection by KR13 and HNG156 were 639 ± 68.9 nM and 5.5 ± 0.9 µM, respectively. Gp120 Retention (b) was tested by western blot analysis using D7324 anti-gp120 on the residual gp120 on the virions treated with KR13. The IC_50_ of KR13 gp120 shedding was 1.2± 0.93 µM. Release of p24 by KR13 and HNG156 (c) was tested with a p24 leakage assay using ELISA as explained in the Materials and Methods section. The IC_50_ value for KR13 induced virolysis was 4.4 ± 0.7 µM. No p24 release was observed by HNG156 upto at 200 µM. All of the above EC_50_ and IC_50_ values were obtained using the Origin Pro. 8 software with sigmoidal fits. Error bars represent the standard deviation of the mean, n = 3.


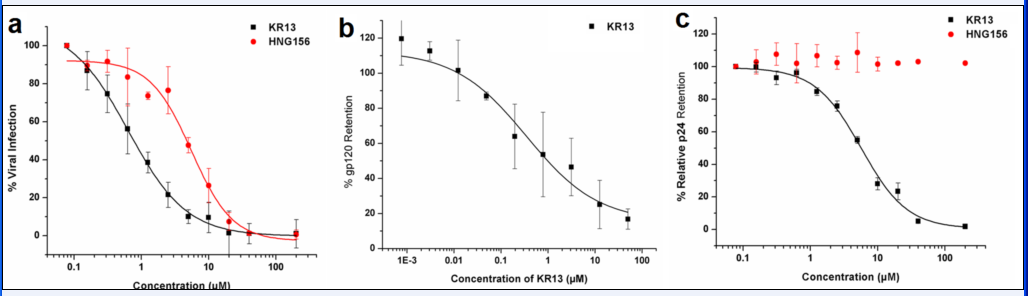


**Figure S13:** Dose responses of the effects of peptide triazoles, KR13 and HNG156 on HIV-1 BaL pseudovirus induced infection inhibition of HOS CD4^+ve^ CCR5^+ve^ and HOS CD4^-ve^ CCR5^+ve^ cells. Extent of cell infection was measured using a single round infection assay. Origin Pro. 8 was used for sigmoidal curve fitting, n=3. The x-axis represents Relative Luminescence Units (RLU) which was calculated by subtracting the background signal (approximately 120 RLU).





1. Tuzer F, Madani N, Kamanna K, Zentner I, LaLonde J, Holmes A, Upton E, Rajagopal S, McFadden K, Contarino M *et al*: **HIV-1 Env gp120 structural determinants for peptide triazole dual receptor site antagonism**. *Proteins* 2013, **81**(2):271-290.
